# Bayesian Two-Process Weibull Modelling of PCL-Blend-PEG Coating Degradation in Simulated Body Fluid: Composition-Held-Out Validation, Identifiability Limits and Exploratory Electrochemical Correlation

**DOI:** 10.3390/polym18182237

**Published:** 2026-09-14

**Authors:** Vlad-Cristian Stoica, Anita Ioana Visan

**Affiliations:** 1 Independent Researcher, 031777 Bucharest, Romania; stoicavladcristian05@gmail.com; 2 Lasers Department, National Institute for Lasers, Plasma and Radiation Physics, 409 Atomistilor Street, Magurele, 077190 Ilfov, Romania

**Keywords:** polycaprolactone, poly(ethylene glycol), polymer blend coating, degradation kinetics, Weibull model, Bayesian inference, leave-one-composition-out validation, parameter identifiability, small-data machine learning, simulated body fluid

## Abstract

Predicting how polycaprolactone–poly(ethylene glycol) (PCL-blend-PEG) implant coatings lose mass in simulated body fluid (SBF) would shorten costly 16-week immersion campaigns, but published studies typically provide only three to five compositions, too few for flexible data-driven models. We re-analysed the 16-week mass-loss curves of dip-coated PCL, PCL-blend-PEG 3:1, PCL-blend-PEG 1:3, and PEG films on titanium using a fully specified Bayesian workflow. A mechanistically motivated two-process Weibull model, combining a fast PEG dissolution term and a slow PCL hydrolysis term with composition-dependent time scales, was compared against a parsimonious single effective-Weibull baseline under a prespecified decision rule with exact leave-one-composition-out (LOCO) and future-time refits. Both models converged cleanly (maximum $\hat{R}\leq 1.005$, zero divergences), yet the two-process model did not outperform the baseline: pooled leave-one-composition-out mean absolute error was 43.7 percentage points for the two-process model versus 37.6 for the baseline, and pooled temporal error 13.2 versus 11.8 percentage points, respectively. The PEG parameters of the two-process model were strongly correlated (r=0.956), precluding separate identification of PEG and PCL kinetics. Empirical coverage of the nominal 90% LOCO intervals was only 21.4% for the two-process model and 3.6% for the baseline. Composition-wise, residuals localise the failure: PEG release inside blends is matrix-retarded rather than instantaneous. We report the negative result transparently, quantify the additional data needed, and release the full reproducible pipeline. These findings define the current predictive limits imposed by the available experimental data and provide guidance for future experimental design.

## 1. Introduction

Biodegradable polymer coatings are a practical route to functionalising metallic orthopaedic and dental implants: they provide a resorbable reservoir for drugs or growth factors, tune the initial tissue response, and disappear once osseointegration is established, and they can simultaneously improve the corrosion resistance of the underlying metal [1]. Poly(ε-caprolactone) (PCL) is the workhorse of this class because it is semicrystalline, ductile, inexpensive, approved for clinical use, and hydrolyses slowly enough to survive months in physiological media [2,3]. However, its slow hydrolysis is also its main limitation: unmodified PCL barely erodes over the time scale of early bone healing, and its hydrophobicity limits the release of hydrophilic payloads [4,5]. Blending PCL with poly(ethylene glycol) (PEG) is the standard remedy. Two behaviours must be distinguished here. Pure PEG, in the form of a stand-alone film immersed in an aqueous medium, is highly water-soluble and dissolves almost immediately, on a minute time scale. Blend PEG, that is, PEG dispersed inside a PCL matrix, does not have the same immediate access to bulk water: its release depends on connectivity to the external solution, water ingress, and the concurrent evolution of the surrounding PCL matrix, and it leaves behind a porous PCL skeleton whose permeability, wettability, and erosion rate scale with the PEG fraction that was originally present [6,7]. Throughout this manuscript, we therefore reserve ‘pure PEG’ for the c = 0 coating and use ‘blend PEG’ when we refer to the PEG contained inside a PCL-blend-PEG film. In a previous study, we fabricated PCL, PCL-blend-PEG (3:1 and 1:3, *w*/*w*), and PEG coatings on titanium by dip coating and followed their degradation in simulated body fluid (SBF) for up to 16 weeks, complementing the gravimetric measurements with FTIR, GIXRD, SEM, wettability, and potentiodynamic polarisation analyses [8]. The qualitative conclusion was unambiguous: PEG solubilises within minutes, PCL degrades over months, and blend degradation increases monotonically with PEG content. However, that study did not deliver a predictive description. Two questions remained open, and they are the questions that matter for coating design: (i) can the mass-loss trajectory of an untested blend composition be forecast from a small number of measured compositions, and (ii) can the contributions of PEG dissolution and PCL hydrolysis be separated quantitatively from mass-loss data alone?

Both questions are attractive targets for machine learning, and the polymer-informatics literature now offers many examples of neural networks predicting polymer properties [9,10]. The difficulty is data volume. Degradation studies of coated implants are labour-intensive; a 16-week immersion series with weekly gravimetry on a handful of compositions is a substantial experimental effort, yet it yields only tens of data points spanning three to five composition levels. Under such conditions, flexible models overfit catastrophically, and the small-data literature in materials science consistently recommends embedding domain knowledge, restricting model capacity, or moving to explicitly probabilistic formulations rather than increasing network depth [11,12,13,14]. Consistent with this literature, an initial attempt to fit an artificial neural network to the same dataset in Mathematica did not produce a validated predictor; the technical details of that attempt, and the reasons it could not be used as a comparator, are documented in Section 2.9 and Section 3.12. The present study therefore adopts a domain-constrained probabilistic approach rather than a network-based one.

We therefore replaced the neural network with a mechanistically constrained Bayesian kinetic model. The Weibull (stretched-exponential) function is the natural building block: it is the standard empirical descriptor of polymer erosion and matrix release; it interpolates between diffusion-controlled and relaxation-controlled regimes through a single shape parameter, and its parameters carry a direct kinetic reading [15,16,17]. Because PEG dissolution and PCL hydrolysis are physically distinct processes with time scales separated by three orders of magnitude, a two-process Weibull mixture weighted by the nominal blend composition is the obvious mechanistic candidate. Casting it in a Bayesian framework adds three features that a least-squares fit cannot provide: honest predictive intervals for unmeasured compositions, explicit diagnosis of which kinetic parameters the data can and cannot determine, and a principled comparison against a deliberately simpler alternative [18,19,20,21,22,23].

The contribution of this paper is deliberately framed around what the data can support. We (i) reconstruct and audit a fully documented 28-point degradation dataset with declared provenance and uncertainty status; (ii) specify a two-process Weibull kinetic model and a single effective-Weibull baseline with priors fixed before any held-out evaluation; (iii) evaluate both under exact leave-one-composition-out (LOCO) and future-time refits, so that no held-out composition or time point can leak into training; (iv) apply a prespecified selection rule combining held-out error, convergence diagnostics and identifiability screens; (v) localise the residual structure that explains the outcome; and (vi) explore, descriptively, whether the fitted kinetic time scale tracks the electrochemical parameters measured on the same coatings [8]. The headline result is negative: the mechanistically richer model does not earn its extra parameter, and neither model extrapolates reliably to an unseen composition. We argue that publishing this result with its diagnostics is more useful to the field than publishing an unvalidated predictor, and we quantify the experimental design that would be required to change the conclusion [24,25].

## 2. Materials and Methods

### 2.1. Source Experimental Data

All degradation observations analysed here originate from the immersion experiments reported in our earlier study [8] and are described there in full. Briefly, PCL ($M_{n}=14,000\;Da$), PEG ($M_{n}=6$ kDa), and PCL-blend-PEG mixtures at 3:1 and 1:3 mass ratios were dip-coated onto polished titanium substrates and immersed in simulated body fluid prepared according to the established protocol for bioactivity assessment [26] at 37 °C under dynamic flow (1.31 ± 0.04 mL min^−1^ per channel, two samples per channel, three independent weight determinations per point). Relative mass loss was recorded at 2, 4, 8, 10, 12, 14, and 16 weeks for PCL and the two blends, and at 1, 3, 9, 12, 48, 60, and 120 min for PEG, whose dissolution is complete within two hours. Throughout this paper, compositions are indexed by the PCL mass fraction c, so that c=0 (PEG), c=0.25 (PCL-blend-PEG 1:3), c=0.75 (PCL-blend-PEG 3:1), and c=1 (PCL). No new immersion, gravimetric, or electrochemical experiments were performed for the present work; this is a modelling and validation study. A clarification of the source-material provenance is warranted. As explicitly stated in Section 2.1 of the source publication [8], the PCL used in the original immersion experiments had a number-average molecular weight of 14,000 Da, and the PEG had a number-average molecular weight of 6000 Da; both polymers were supplied in powder form by Sigma-Aldrich (Darmstadt, Germany) and used without chemical modification. The two polymers were dissolved in chloroform (Merck, Darmstadt, Germany, purity 99%) at a total concentration of 30 g L^−1^ prior to dip-coating on grade-4 commercial-purity titanium discs of 12 mm diameter and 0.2 mm thickness, at a withdrawal velocity of 100 mm min^−1^; the resulting coating thickness measured by step profilometry ranged from 0.6 to 2 μm depending on composition [8]. Because the present study is a Bayesian reanalysis of the mass-loss curves published in [8], batch-specific parameters such as residual catalyst content from ring-opening polymerization of PCL, initial acid-end concentration, and initial water content are not disclosed in [8] and are therefore not accessible to the present model; their effects are absorbed, together with all other unmodelled sources of variability, into the calibrated Weibull parameters and the noise scale. This constraint of the source-data provenance is now made explicit in Section 4.7 (Limitations) and further discussed in Section 4.8.

### 2.2. Dataset Reconstruction and Audit

Replicate-level masses and numerical standard deviations were not preserved in tabular form, and the mass-loss curves exist only as a raster figure in the source publication [8]. We therefore reconstructed the dataset by axis-calibrated digitisation of every marker centre in that figure, yielding 28 observations (seven time points × four compositions). Pixel coordinates, axis calibration points, and the resulting conversion are archived in the repository, together with an overlay figure that superimposes the selected centres on the source raster (Figure S1). Practical centre-reading uncertainty is approximately ±0.3 percentage points, rising to ±0.5 percentage points where blend markers overlap. Because graph-reading uncertainty is not an experimental standard deviation, no observation-level standard deviation was entered into the likelihood, and every observation is flagged provisional = true in the released dataset.

Three classes of numerical conflict were identified and resolved by a single prespecified rule: retain the plotted marker centre, record the discrepancy, and correct nothing silently. First, the source text quotes “almost 82%” for the 3:1 blend at 16 weeks whereas the plotted marker reads 76.5%; second, it quotes 98% for PEG at 120 min whereas the plotted marker reads 95.4%; third, the legacy Mathematica notebook contains hard-coded values that place 4.5% mass loss at 2 weeks instead of 4 weeks for PCL and assign 82% loss to pure PCL at 16 weeks, against a plotted value near 21%. All notebook literals were rejected because they conflict with the figure and lack traceable provenance; the complete candidate-by-candidate audit is provided as Supplementary Table S1. The printed mass-loss definition in the source publication, $[(m_{f}-m_{i})/m_{i}]\times 100\%$, carries a sign inconsistent with the positive plotted curves; we interpret the reported quantity as relative mass loss, $[(m_{i}-m_{f})/m_{i}]\times 100\%$, and flag the typographical issue rather than propagating it.

### 2.3. Kinetic Models

Let c∈[0,1] denote the PCL mass fraction, x=c−0.5 the centred composition, and t the immersion time in hours. The **two-process Weibull model** treats PEG dissolution and PCL hydrolysis as parallel, composition-weighted processes:

$$log\tau_{PEG}(c)=\alpha_{PEG}+\gamma_{PEG}\;x,\;\;log\tau_{PCL}(c)=\alpha_{PCL}+\gamma_{PCL}\;x$$

$$F_{j}(t,c)=1-exp\;\left[-{\left(\frac{t}{\tau_{j}(c)}\right)}^{\beta_{j}}\right],\;\;j\in \{PEG,PCL\}$$

$$\mu (c,t)=100\left[\left(1-c)\;F_{PEG}(t,c)+c\;F_{PCL}(t,c\right)\right]$$

The Weibull fraction is evaluated on the log-time scale for numerical stability and $F_{j}(0)=0$ is imposed exactly. This construction guarantees three physical invariants without any post hoc clipping: zero mass loss at t=0, a latent response confined to [0,100]%, and monotone non-decreasing loss in time. The shape parameters $\beta_{j}$ are shared across compositions, whereas both time scales vary continuously with composition through the slopes $\gamma_{j}$; a positive $\gamma_{PCL}$ therefore encodes the expected retardation of hydrolysis in PCL-rich films.

The **effective-Weibull baseline** deliberately abandons process separation. Pure PEG is described by its own Weibull curve, and every PCL-containing material shares a single effective Weibull whose log time scale is linear in centred composition:

$$log\tau_{eff}(c)=\alpha_{eff}+\gamma_{eff}\;x\;(c>0),\;\;\mu (c,t)=100\left[1-exp\;\left(-{\left(t/\tau (c)\right)}^{\beta }\right)\right]$$

The baseline is intentionally piecewise at c=0, reflecting the three-order-of-magnitude gap between the PEG and PCL experimental windows. It carries seven stochastic parameters against eight for the two-process model, so the additional process separation must earn its extra complexity.

### 2.4. Observation Model and Priors

Because provisional digitised data can contain outliers that Gaussian errors would over-weight, the primary observation model is a correctly normalised Student-*t* distribution truncated to [0,100]%, with location μ(c,t), scale σ and degrees of freedom ν. Priors were fixed from published PEG-dissolution and PCL-hydrolysis time scales before any held-out fold was evaluated and were never tuned against validation outcomes (Table 1). A non-truncated Student-*t* likelihood was retained only as an explicit sensitivity analysis.

Prior predictive simulation was performed on both linear and logarithmic time axes before inference and is reported in Figure S2. Prior plausibility was judged on whether simulated curves spanned the physically admissible range without concentrating on degenerate trajectories, not on whether they resembled the observations.

### 2.5. Posterior Inference and Convergence Criteria

Posteriors were sampled with the No-U-Turn Sampler [18] through PyMC [19,20] using the compiled nutpie backend, with four chains, 2000 tuning iterations and 2000 retained draws per chain (8000 posterior draws), target acceptance 0.95 and maximum tree depth 15. All random seeds derive deterministically from a single master seed (20200324), and computation used float64 throughout. Convergence was assessed with rank-normalised split-$\hat{R}$, bulk and tail effective sample size (ESS), divergent-transition counts, tree-depth saturation and the Bayesian fraction of missing information (BFMI), using ArviZ [21,27]. Acceptance required $\hat{R}<1.01$, bulk and tail ESS ≥400, zero divergences, no tree-depth saturation and BFMI ≥0.30; a missing criterion was never treated as a pass.

### 2.6. Held-Out Validation Design

Two validation schemes were specified before fitting, and in both cases the model was refitted from scratch on the reduced training set rather than conditioned post hoc.

Leave-one-composition-out (LOCO). Four folds hold out all seven observations of one composition: fold A (c = 0), B (c = 0.25), C (c = 0.75), D (c = 1). Folds B and C are composition interpolations; A and D are boundary extrapolations. Fold constructors assert programmatically that no observation of the held-out composition can enter training. No observation-level random split was used because random splitting across a curve leaks the curve itself.

Future-time forecasting. PCL-containing materials were fitted using observations up to and including 8 weeks (1344 h) and asked to predict the exact 10-, 12-, 14-, and 16-week points; PEG was fitted through 60 min and asked to predict the exact 120 min point.

Reported metrics are mean absolute error (MAE), root-mean-square error (RMSE), median and maximum absolute error, endpoint error, exact posterior-mean log predictive density, empirical coverage of nominal 50% and 90% predictive intervals, and mean interval width. Errors are expressed in percentage points (pp) of mass loss.

### 2.7. Prespecified Model-Selection Rule

To exclude retrospective rationalisation, the decision rule was fixed in advance. The two-process model is preferred over the baseline only if **all** of the following hold: clean convergence diagnostics; at least three of four LOCO folds won on MAE; at least 5% relative improvement in both pooled LOCO MAE and RMSE; no worsening of pooled temporal MAE or RMSE; and no serious identifiability caution. Training-set fit was explicitly excluded from the rule. Log predictive density, interval coverage, interval width, and posterior-predictive plots were recorded as contextual checks rather than as thresholds.

### 2.8. Identifiability and Sensitivity Analysis

Eighteen prespecified posterior relationships for the two-process model (and eleven for the baseline) were screened for Pearson correlation exceeding |r|=0.90, including each intercept–slope pair, each shape parameter against its own log time scale at five compositions, and σ against every kinetic parameter [23]. Each marginal posterior was additionally screened for being nearly prior-identical, boundary-pressed, extremely broad, multimodal, or prior-sensitive; a narrow marginal was never reported as accurate when any screen was triggered. Five prespecified sensitivity analyses were attempted: wider and narrower α priors, unconstrained versus positive-only slopes, truncated versus non-truncated Student-*t* likelihood, narrower β prior width, and exclusion of provisional observations. Variants that failed to converge were excluded from robustness claims rather than reported as agreement.

### 2.9. Reference Neural-Network Attempt

For completeness, we document the approach the present workflow replaced. A feed-forward network was implemented in Mathematica on the same four compositions, with the training set expanded to 60 rows by random convex interpolation between measured compositions. Inspection of the saved notebook shows that NetChain, NetInitialize, and NetTrain all failed, that the printed performance metrics remained unevaluated symbolic expressions, that the augmented rows leak interpolated targets into training, and that the reported “50:50 prediction” is the unchanged input feature vector rather than a network output. No metric, interval, or prediction from that attempt is reused anywhere in this paper; it is reported only as a documented negative control for the small-data regime.

### 2.10. Exploratory Electrochemical Correlation

Potentiodynamic polarisation parameters for the same four coatings—corrosion potential $E_{corr}$, corrosion current density $i_{corr}$, polarisation resistance $R_{p}$ (Stern–Geary), protective efficiency $P_{e}$ and coating porosity P—were taken from Table 2 of the source publication [8]. Each coating was assigned two model-derived descriptors: the posterior-mean effective time scale ${log}_{10}\tau_{eff}$ and the posterior-mean shape β. Associations were quantified by Spearman rank correlation with exact permutation *p*-values over all 4!=24 rank assignments. With n=4, the smallest attainable two-sided *p*-value is 0.083, so this analysis is explicitly descriptive and hypothesis-generating; it cannot establish significance and is not part of the model-selection rule.

### 2.11. Software, Reproducibility, and Data Availability

The workflow is implemented as five sequential, independently runnable stages (file inventory, dataset construction, model fitting, validation, report generation) in Python 3.12.10 with PyMC [20], ArviZ [27], NumPy, pandas, SciPy, and Matplotlib. Automated tests (47 in total) pin the SHA-256 hashes of the scientific source files, assert the physical invariants of both kinetic models, verify unit conversions and fraction bookkeeping, enforce grouped-split integrity, and check that the pure-PEG endpoint is treated as 120 min and never as 16 weeks. Every posterior summary, diagnostic table, validation prediction, and figure in this paper is regenerated by the released pipeline from the released dataset, in line with FAIR data practice [24] and with transparent-reporting recommendations for predictive models [25]. In line with journal policy on generative artificial intelligence, we note that the analysis code, the report generation, and the manuscript text were produced with the assistance of an agentic large-language-model tool (Perplexity Computer, 2026) working from specifications set by the authors; the model formulation, priors, and prespecified decision rule were authored and approved by the authors, and every reported number was verified by re-executing the released pipeline (see Acknowledgments for the full disclosure).

## 3. Results

### 3.1. Reconstructed Dataset

**Figure 1:**
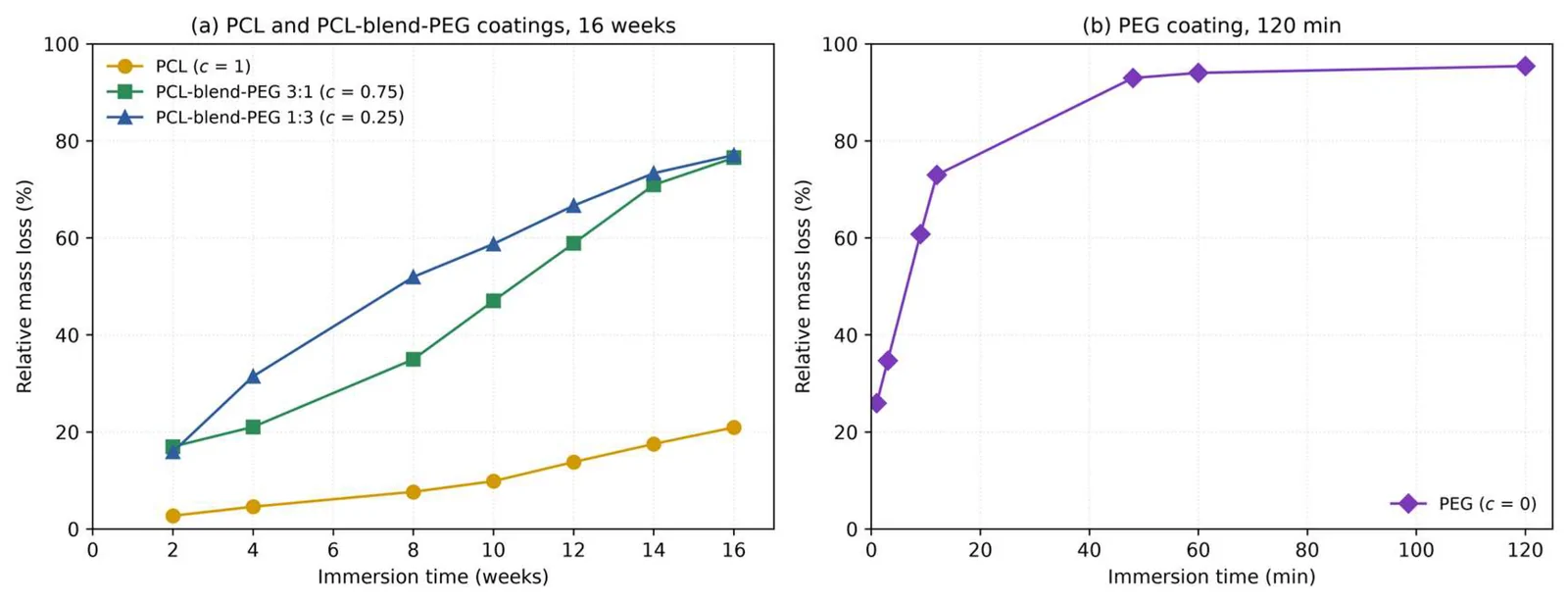
Reconstructed degradation dataset used for inference, digitised from Figure 6 of [8]: (**a**) PCL and PCL-blend-PEG coatings over 16 weeks; (**b**) PEG coating over 120 min. Note the three-order-of-magnitude difference in the time axis. All points are provisional digitisations; connecting lines are guides to the eye only.

The audited dataset comprises 28 observations across four compositions (Figure 1, Table 2). All 28 are provisional digitisations; none is a raw replicate measurement, and every one of the 28 mass-loss values used in this study was recovered from Figure 6 of the published source paper [8] rather than remeasured in this work. The qualitative behaviour reported originally [8] is reproduced exactly: PEG loses 26% of its mass within the first minute and 95.4% within 120 min; PCL loses only 20.9% over 16 weeks; and the two blends lose 76.5% (3:1) and 77.1% (1:3) over the same period, with the PEG-rich blend degrading faster at every intermediate time. Notably, the two blend curves converge at 16 weeks despite differing threefold in PEG content, which already signals that blend degradation is not a simple composition-weighted superposition of the two pure-component curves.

### 3.2. Convergence

Both full-data fits satisfied every prespecified convergence criterion (Table 3). Maximum $\hat{R}$ was 1.005 for the two-process model and 1.002 for the baseline, minimum bulk ESS 954 and 1577, minimum tail ESS 1719 and 1869, with zero divergent transitions, no tree-depth saturation and BFMI of 0.68 and 0.79 respectively. All ten exact LOCO and temporal refits also converged cleanly (maximum $\hat{R}\leq 1.004$, zero divergences; Table S2). Convergence is therefore not a candidate explanation for any of the predictive failures reported below.

### 3.3. Posterior Kinetic Parameters

Posterior summaries are given in Table 4. The two-process model recovers the expected three-order-of-magnitude separation of time scales: at c=0.5 the posterior-mean PEG time scale is $\tau_{PEG}=exp(-1.006)\approx 0.37$ h (about 22 min), against $\tau_{PCL}=exp(8.50)\approx 4.9\times 10^{3}$ h (about 29 weeks). The PEG shape parameter is close to unity ($\beta_{PEG}=0.99$, 94% HDI 0.36–2.19), consistent with first-order dissolution, whereas the PCL shape parameter exceeds unity ($\beta_{PCL}=2.32$, 94% HDI 0.85–3.83). Mechanistically, β > 1 in a Weibull kinetic law corresponds to a mass-loss rate that increases with time rather than decays: this is the signature expected when hydrolysis is initially rate-limited by water ingress into the still-dense film and only later, once water has fully penetrated and hydrolytic scission has generated carboxylic-acid chain ends, does the bulk hydrolysis rate rise via well-documented acid autocatalysis of ester cleavage [5,28]. The 94% HDI stretches from 0.85 (nearly first-order behaviour) to 3.83 (a strongly delayed, sharply accelerating profile) because 16 weeks of PCL data are not sufficient to pin down the exponent tightly; the posterior mean is consistent with an accelerating profile (β > 1), although the broad 94% HDI includes β = 1, consistent with an induction period before bulk hydrolysis accelerates—the sigmoidal signature expected for slow water uptake followed by autocatalytic chain scission [5,28]. Both composition slopes are positive in the mean ($\gamma_{PEG}=1.78$, $\gamma_{PCL}=0.54$), indicating retardation of both processes in PCL-rich films, but both credible intervals comfortably include zero.

The baseline is markedly better determined. Its effective log time scale has a posterior mean of 7.878 with a standard deviation of only 0.187, and its composition slope is $\gamma_{eff}=1.809\pm 0.530$ with a 94% HDI of 0.902–2.785 that excludes zero. In other words, the aggregate dependence of degradation rate on composition is identified by these data, whereas the decomposition of that dependence into separate PEG and PCL contributions is not.

### 3.4. In-Sample Behaviour and Composition-Wise Residual Structure

**Figure 2:**
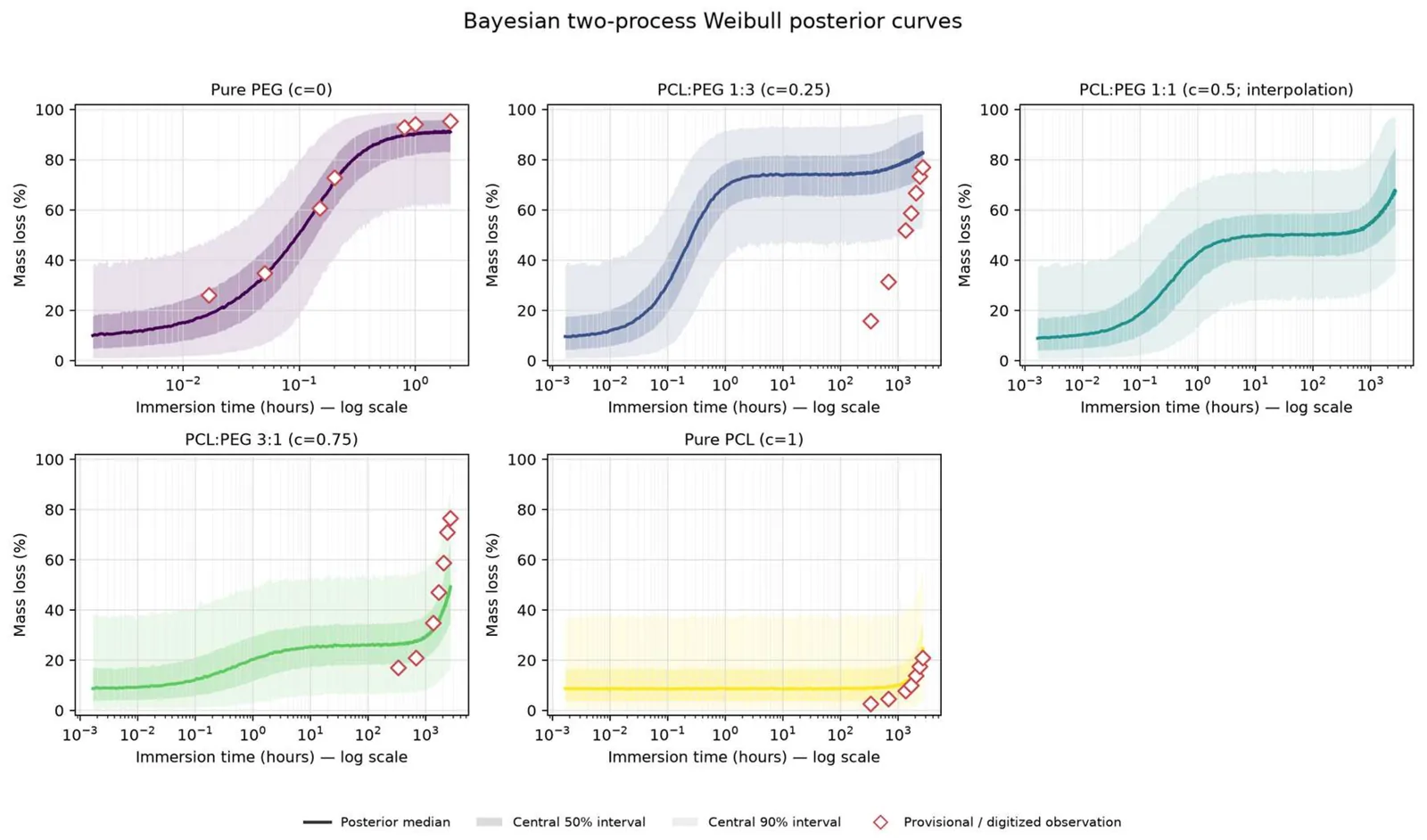
Posterior predictive curves of the two-process Weibull model on a logarithmic time axis, with posterior median, central 50% and 90% predictive intervals, and the provisional digitised observations. The c=0.5 panel is a composition interpolation of the fitted model, not a measurement.

**Figure 3:**
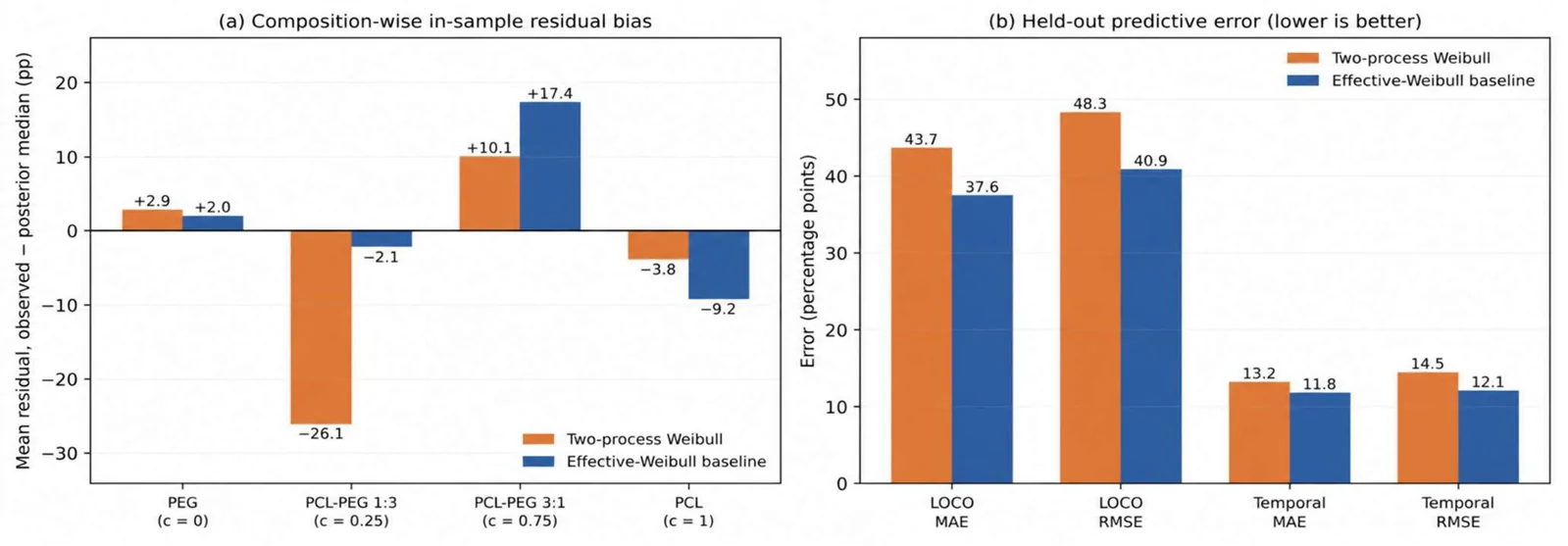
(**a**) Composition-wise mean in-sample residual (observation minus posterior median, percentage points) for both models; the large negative bias of the two-process model at c=0.25 reflects its assumption of near-instantaneous PEG release inside blends. (**b**) Pooled held-out predictive error under exact leave-one-composition-out and future-time refits; lower is better.

Posterior predictive curves for the two-process model are shown in Figure 2. The pure-PEG panel is described well: the fitted curve passes through all seven dissolution points and the 50% band contains them. The blend panels are not. Composition-wise mean residuals (Figure 3a, Table 5) localise the problem precisely. For the PEG-rich blend (c = 0.25), the two-process model over-predicts mass loss by 26.1 pp on average, with a maximum in-sample discrepancy of 59.1 pp; for the PCL-rich blend (c = 0.75), it under-predicts by 10.1 pp. The baseline shows a much smaller and differently signed bias pattern (−2.1 pp at c=0.25, +17.4 pp at c=0.75).

This residual signature has a direct mechanistic reading. The two-process model assumes that the PEG contained in a blend dissolves on essentially the same fast time scale as pure PEG, so that a c=0.25 film should shed close to 75% of its mass within the first hours of immersion and then plateau. The measurements show a gradual rise from 15.9% at 2 weeks to 77.1% at 16 weeks instead. PEG release inside a PCL matrix is therefore matrix-retarded: it is limited by water ingress and by percolation of the PEG domains through the PCL skeleton, not by the intrinsic solubility of PEG. The model’s failure mode is thus informative rather than merely disappointing, and it falsifies a specific, plausible mechanistic assumption.

### 3.5. Leave-One-Composition-Out Validation

**Figure 4:**
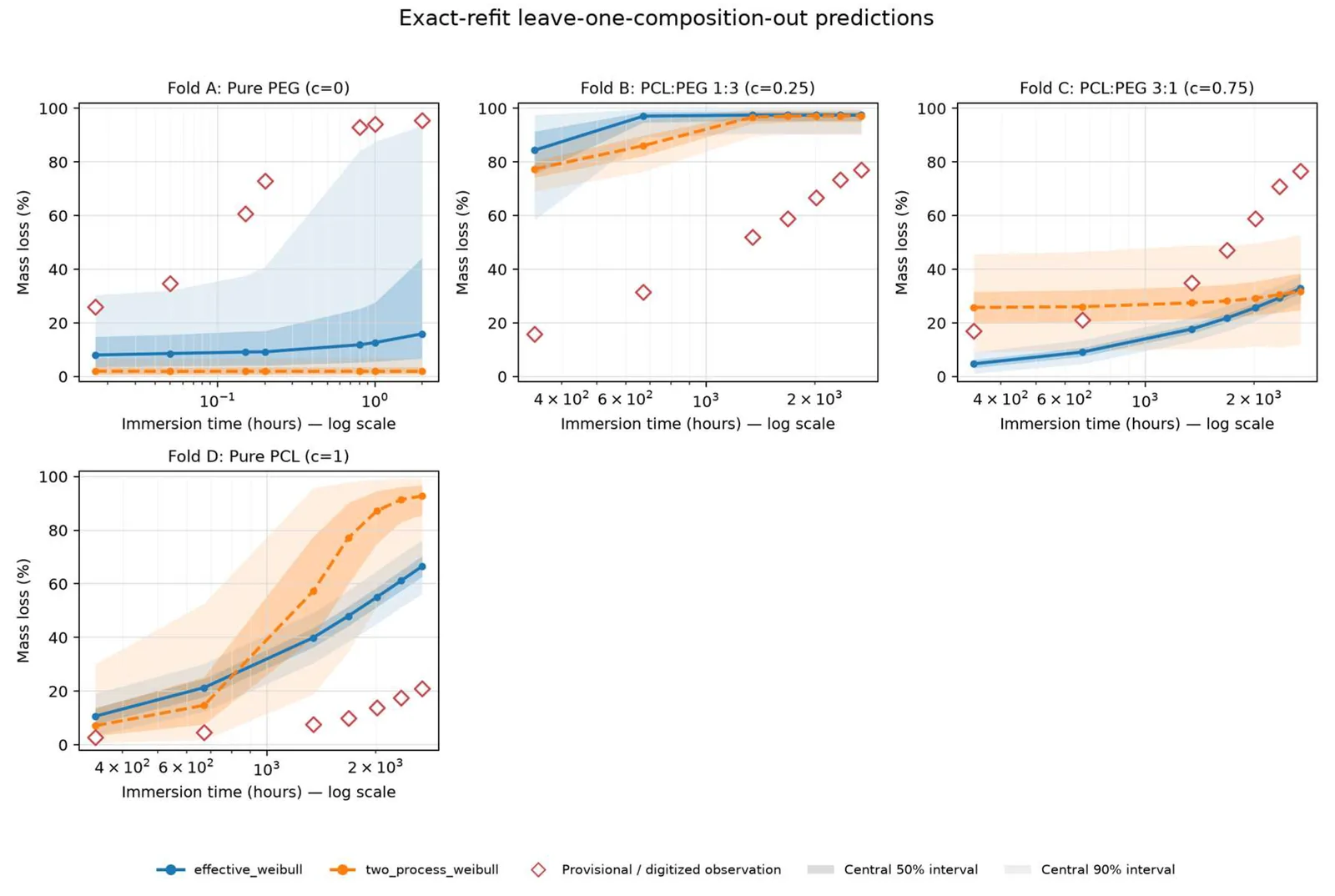
Exact leave-one-composition-out predictions. Each panel shows the posterior predictive median and central 50%/90% intervals obtained after refitting both models with all observations of that composition removed, against the held-out observations. Note the catastrophic and overconfident extrapolation in folds A and D.

**Figure 5:**
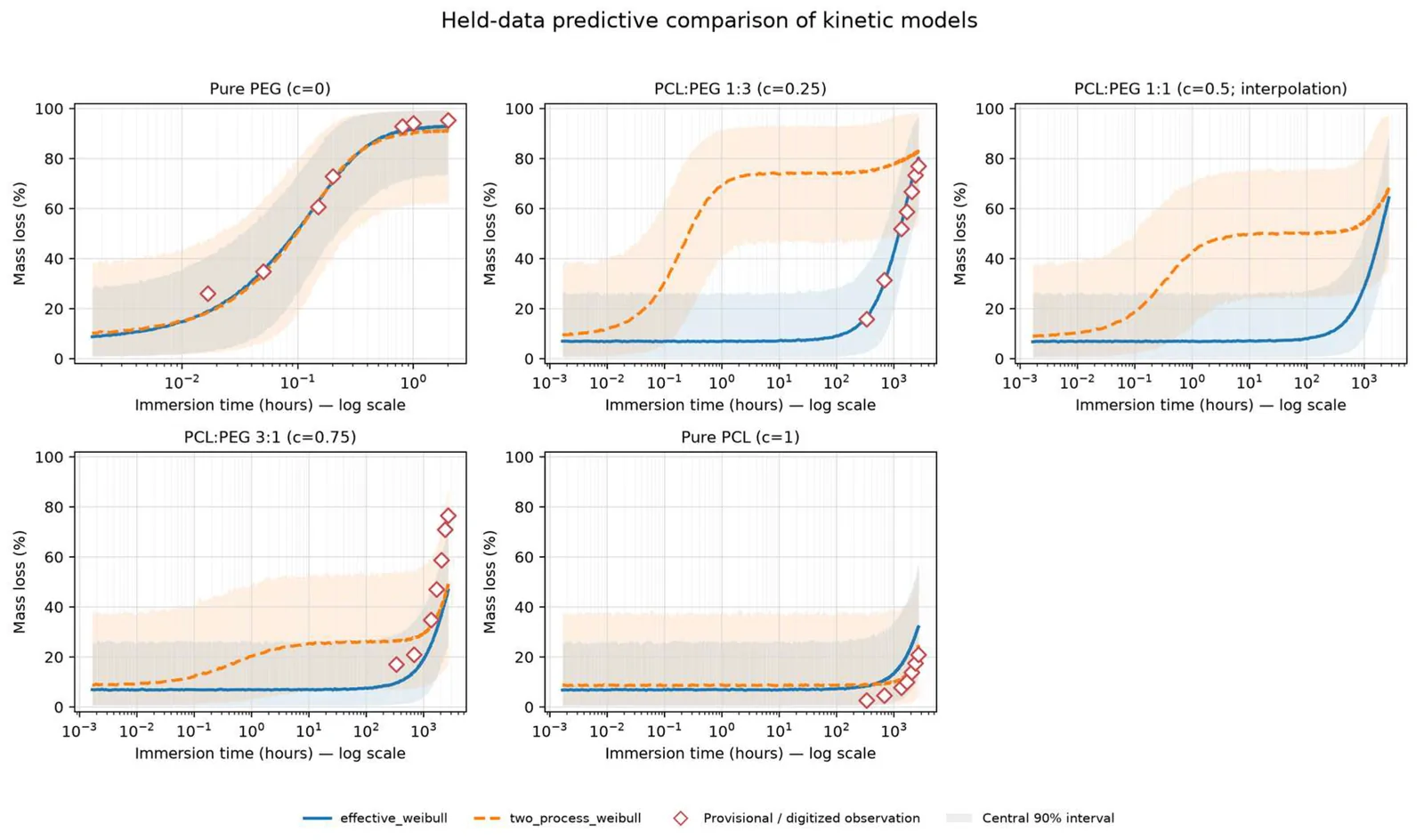
Full-data posterior median curves of the two models over the measured composition range, with 90% predictive intervals. The two models agree closely for pure PEG and diverge strongly for the blends, where the two-process model front-loads mass loss into the first hours of immersion.

Held-out results are reported in Table 6 and Figure 4; the corresponding predictive curves of both models over the whole composition range are compared in Figure 5. Pooled over all 28 held-out observations, the two-process model achieves MAE 43.74 pp and RMSE 48.35 pp, against 37.58 pp and 40.92 pp for the baseline—that is, the mechanistically richer model is 16.4% worse in MAE and 18.2% worse in RMSE. It wins two of four folds (B and C, both interpolations) and loses both boundary-extrapolation folds decisively: on fold A (predicting PEG from PCL-containing films only) its MAE is 65.6 pp against 50.8 pp, and on fold D (predicting PCL from PEG-containing films only) 48.9 pp against 32.2 pp.

Interval calibration is worse than the point errors alone suggest. Empirical coverage of the nominal 90% predictive interval, pooled over folds, is 21.4% for the two-process model and 3.6% for the baseline; nominal 50% coverage is 3.6% and 0%. In fold A, the two-process model produced 90% intervals of average width 6.7 pp around predictions that were wrong by up to 92.9 pp—a textbook case of a converged model being confidently wrong on an extrapolation it has no information about. Neither model is therefore usable for quantitative prediction at an unmeasured composition, and the baseline’s advantage in point error does not make it a validated kinetic law: it is merely the less-poor comparator.

### 3.6. Future-Time Forecasting

**Figure 6:**
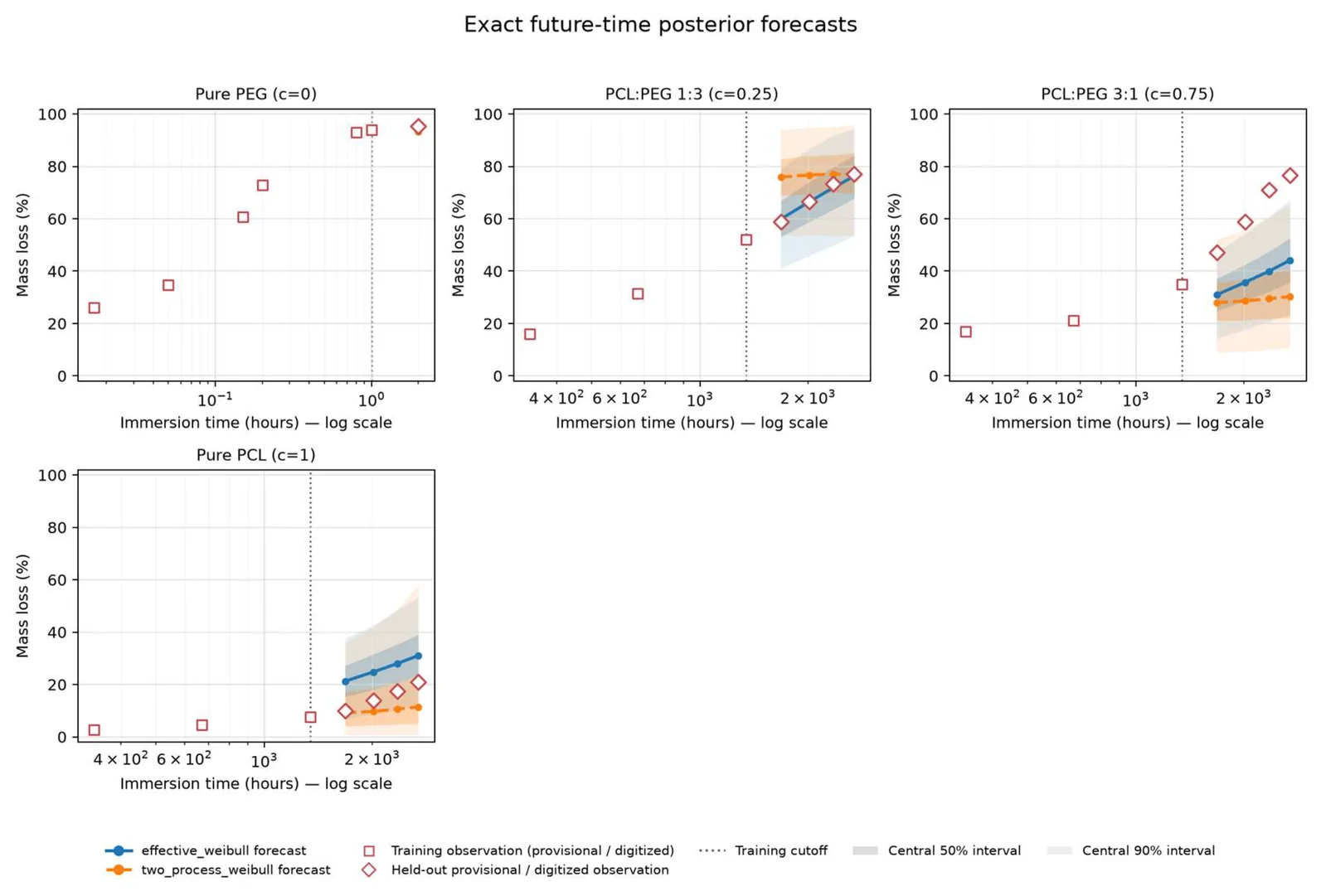
Exact future-time posterior forecasts. Squares are training observations, diamonds are held-out later observations, and the dotted line marks the training cut-off. Temporal extrapolation over one additional experimental octave is tractable; extrapolation to an unmeasured composition (Figure 4) is not.

Extrapolation in time is far more benign than extrapolation in composition (Table 7, Figure 6). Fitting only observations up to 8 weeks, the two-process model predicts the four later PCL points with MAE 2.03 pp and full nominal coverage, and the four later c=0.25 points with MAE 7.20 pp. Its weakness is concentrated in the PCL-rich blend, where it predicts a plateau near 30% while the measurements continue to 76.5%, giving MAE 32.36 pp and a 16-week endpoint error of 43.47 pp. The baseline forecasts the same fold better (MAE 25.47 pp) and is markedly better for c=0.25 (MAE 1.35 pp) and for pure PEG (1.67 pp versus 5.74 pp), though worse for pure PCL (11.21 pp versus 2.03 pp). Pooled over all 13 held-out future observations, the baseline again wins: MAE 11.83 pp versus 13.24 pp and RMSE 12.13 pp versus 14.48 pp. Both models therefore violate the “temporal must not worsen” clause of the decision rule in favour of the baseline.

### 3.7. Outcome of the Prespecified Decision Rule

Table 8 evaluates the prespecified rule. Only the convergence clause is satisfied. Fold wins (2/4) fall short of the required 3/4; the relative changes in pooled LOCO MAE and RMSE are −16.4% and −18.2% against a required +5%; pooled temporal MAE and RMSE both worsen by 11.9% and 19.5%; and one required posterior correlation exceeds the identifiability threshold. The available evidence does not support preferring the two-process Weibull model over the single effective-Weibull baseline. We emphasise the converse as well: the baseline passes the comparison only relatively. With 3.6% empirical coverage of its nominal 90% LOCO intervals, it is not a validated predictive law either.

### 3.8. Identifiability

One of eighteen prespecified posterior relationships in the two-process model breaches the correlation threshold, and it is the decisive one: $\alpha_{PEG}$ and $\gamma_{PEG}$ have a Pearson correlation r=0.956 (Table 9, Figure S3). The PEG intercept and its composition slope are thus not separately determined; the data constrain essentially a single linear combination, so $\tau_{PEG}$ can be traded against its composition dependence with almost no change in likelihood. Five of eight parameters trigger at least one caution screen: $\gamma_{PEG}$, $\beta_{PCL}$ and σ are extremely broad, and $\alpha_{PEG}$, $\gamma_{PEG}$, $\gamma_{PCL}$, $\beta_{PCL}$, and σ are prior-sensitive. The baseline breaches no correlation threshold in eleven relationships and triggers cautions on only two parameters (both variance components).

The structural reason is that the experimental design contains exactly one composition at which the fast process can be observed in isolation (c = 0) and one at which the slow process can (c = 1). Two intermediate blends must then support four kinetic parameters plus two composition slopes. This is the familiar non-identifiability of sums of exponential-like terms with unknown rates and amplitudes [23]: convergence diagnostics are silent about it, because the sampler explores the ridge perfectly well.

### 3.9. Prior and Likelihood Sensitivity

Of the five prespecified sensitivity analyses, three are informative, one failed convergence, and one is not estimable (Table 10). Narrowing the α priors, constraining slopes to be positive, narrowing the β prior width and removing the likelihood truncation all leave in-sample MAE within 0.7 pp of the default fit (11.63–12.54 pp), so the reported conclusions are not artefacts of these particular choices. Widening the α prior standard deviation to three produced $\hat{R}=1.737$ and bulk ESS of six even after a documented retry; that variant is excluded from all robustness claims rather than reported as an estimate. The “exclude provisional observations” analysis is not estimable because all 28 observations are provisional digitisations—an honest statement of the ceiling on what this dataset can support.

### 3.10. Interpolation to the Unmeasured 50:50 Composition

Because the 1:1 PCL-blend-PEG composition (c = 0.5) is the intermediate point most relevant to applications targeting balanced degradation and drug-release kinetics [8], we report the model interpolation explicitly and with its full caveat. At 2688 h (16 weeks), the two-process posterior median mass loss at c=0.5 is 67.6%, with a central 50% interval of 54.3–84.9% and a central 90% interval of 36.4–97.3%. This is an interpolation of a constrained model between measured compositions, conditional on the digitised data and the stated priors. It is not a measurement and not external validation, and in light of the LOCO coverage reported in Section 3.5, the nominal 90% interval should be regarded as optimistic. A 61-percentage-point-wide 90% interval is, in practice, the honest answer that this dataset supports for an untested composition.

### 3.11. Exploratory Correlation with Electrochemical Parameters

**Figure 7:**
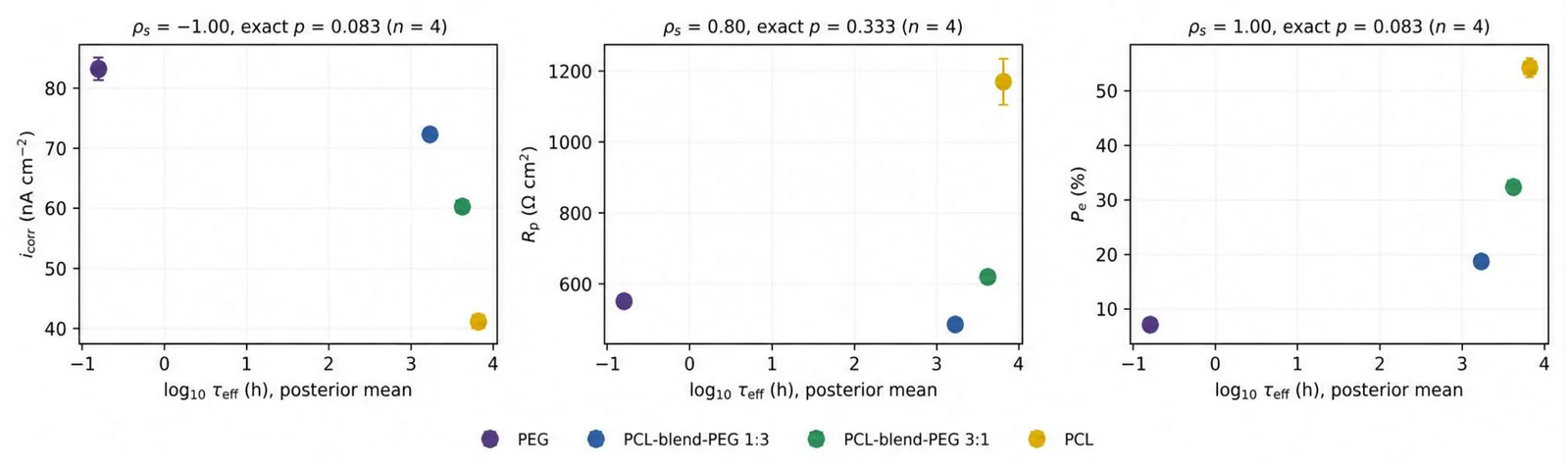
Exploratory association between the posterior-mean effective degradation time scale and three electrochemical parameters measured on the same coatings [8]: corrosion current density, polarisation resistance and protective efficiency. Error bars are the reported measurement standard deviations. Rank correlations are monotone with the physically expected signs but cannot reach significance at n=4.

The electrochemical parameters measured on the same four coatings [8] vary monotonically with the fitted degradation time scale (Table 11, Figure 7). Coatings with a longer effective Weibull time scale have lower corrosion current density (Spearman ρS = −1.00), higher polarisation resistance (ρS = +0.80), higher protective efficiency (ρS = +1.00) and lower porosity (ρS = −0.80). The signs are exactly those expected physically: a slowly degrading, PCL-rich, low-porosity film presents a more continuous barrier to electrolyte ingress, which suppresses the substrate corrosion current and raises the measured protective efficiency [29,30].

Three cautions are essential. First, with n=4, the smallest attainable exact two-sided *p*-value is 0.083, so even a perfect rank correlation is not statistically significant; these associations are descriptive. Second, $\tau_{eff}$ is by construction a monotone function of composition for c>0, so the correlations largely restate the composition dependence of both measurements rather than establishing an independent kinetic–electrochemical link. Third, the electrochemical measurements were made after short exposures whereas the mass-loss time scales are calibrated on the 16-week series, so the two quantities describe different stages of the same process. A genuine test would require potentiodynamic measurements at several immersion times per composition, allowing the electrochemical response to be predicted as a function of accumulated mass loss rather than correlated with a single fitted constant.

### 3.12. The Neural-Network Reference Attempt

The Mathematica neural-network implementation described in Section 2.9 produced no usable output: network construction, initialisation and training all failed, the printed metrics remained unevaluated symbolic expressions, the 60-row training set was generated by convex interpolation between measured compositions (so held-out targets leak into training by construction), and the reported 50:50 “prediction” is the unchanged input feature vector. We report this explicitly because such artefacts are easy to mistake for results: a notebook that prints numbers next to the words “prediction” and “error” looks like a validated model even when nothing was fitted. Under the present design—28 points, four composition levels, no replicate-level noise estimate—no network of practically useful capacity can be trained or honestly validated, which is precisely the regime the small-data materials-informatics literature identifies as requiring domain-constrained or probabilistic alternatives [11,12,13,14].

## 4. Discussion

### 4.1. A Negative Result with Mechanistic Content

The central finding is that a two-process Weibull description, although mechanistically appealing and cleanly sampled, does not outperform a single effective Weibull law on any held-out criterion for this system. That could be dismissed as a failure of model choice, but the composition-resolved residuals (Section 3.4) point to something more specific. The two-process model fails where and how it should fail if its central assumption is wrong: it over-predicts early mass loss in the PEG-rich blend by 26 pp because it treats blend PEG as dissolving on the pure-PEG time scale. The measurements instead show blend degradation proceeding over weeks, with the 1:3 and 3:1 blends converging to almost the same 16-week loss (77.1% versus 76.5%) despite a threefold difference in PEG content.

The physically coherent interpretation is that PEG release from a blend coating is controlled by transport through the PCL matrix rather than by PEG solubility. Below the percolation threshold of the PEG phase, isolated PEG domains can only be reached as water penetrates and as PCL hydrolysis opens connectivity, so PEG leaching becomes coupled to—and rate-limited by—the slow process. This is consistent with reports that porogen leaching from hydrophobic matrices is governed by pore connectivity [7], with PEG-content-dependent erosion in PLGA–PEG systems [6], and with the SEM observations of progressively opening degradation holes in the source study [8]. It also explains the convergence of the two blend curves: once connectivity is established, the accessible fraction saturates, and further loss is dictated by PCL hydrolysis, which is composition-insensitive at these ratios.

A model that encodes this—for instance, a PEG time scale that depends on the degraded state of the PCL phase, or a sequential rather than parallel process coupling—is the natural next candidate. We deliberately did not fit such a model here: it was not prespecified, and testing it on the same 28 points that suggested it would convert a legitimate hypothesis into a post hoc fit. Its evaluation belongs to a study with a purpose-designed dataset.

### 4.2. Why Composition Extrapolation Fails and Time Extrapolation Does Not

The contrast between the LOCO and temporal results (43.7 pp against 13.2 pp pooled MAE) is instructive for anyone planning a predictive degradation study. Forecasting from 8 weeks to 16 weeks asks the model to continue a trajectory it has already observed over a comparable duration, and both models manage this within roughly 12 pp. Predicting an unmeasured composition asks the model to interpolate—or, in folds A and D, extrapolate—a functional dependence on c that is supported by only three remaining levels. With four composition levels in total, removing one destroys a quarter of the design and, at the boundaries, removes the only observation of an entire kinetic regime. Fold A is particularly stark: after removing c=0, no observation shorter than 2 weeks remains anywhere in the training set, so the model is asked to predict minute-scale dissolution having never seen a sub-week measurement.

The consequence is a design recommendation rather than a modelling one. A dataset intended to support composition prediction needs composition levels at a spacing finer than the curvature of the composition–rate relationship, plus at least one interior level held out for genuine validation; five to seven levels (e.g., c=0,0.2,0.4,0.5,0.6,0.8,1) with replicate gravimetry would allow LOCO interpolation folds that are informative rather than degenerate. Equally important, blends should be sampled in the *early* time window (hours to days), not only from 2 weeks onward. The entire early-time behaviour of the blends—precisely the regime where the competing mechanistic hypotheses differ most—is currently unobserved.

### 4.3. Identifiability, and Why Clean Convergence Is Not Evidence

The posterior correlation of 0.956 between $\alpha_{PEG}$ and $\gamma_{PEG}$ deserves emphasis because it is invisible to the diagnostics most commonly reported. Both models achieved $\hat{R}\leq 1.005$ with zero divergences and ESS in the thousands: by conventional standards, exemplary convergence. Yet a two-parameter ridge means the PEG time scale and its composition dependence are not separately estimable, so any claim that this model “quantifies the PEG contribution to blend degradation” would be unsupportable regardless of how well it fitted. This is the classic non-identifiability of superposed exponential-like kinetics with unknown rates and amplitudes [23], and it argues for reporting parameter-level identifiability screens alongside sampler diagnostics as a matter of routine. Notably, the parsimonious baseline does identify what the data can support: its composition slope $\gamma_{eff}=1.81$ with a 94% HDI of 0.90–2.79 is a defensible quantitative statement that degradation slows by roughly a factor of $e^{1.81}\approx 6$ per unit increase in PCL fraction, even though the underlying mechanism remains unresolved.

### 4.4. Interval Calibration as the Primary Practical Failure

For coating design, the decisive number is not the point error but the coverage: nominal 90% intervals that contain the truth 21.4% (two-process) or 3.6% (baseline) of the time on held-out compositions. Point predictions that are wrong by 40 pp are recognisably useless; over-confident intervals that are wrong by 40 pp are dangerous, because they invite a decision. Fold A illustrates the pathology: 90% intervals averaging 6.7 pp in width around predictions in error by up to 92.9 pp. The mechanism is straightforward—with the entire fast-kinetics regime removed from training, the posterior over $\tau_{PEG}$ collapses onto a region determined by the prior and by the blends, and the resulting predictive distribution is narrow for the wrong reason. Any predictive claim about polymer coating lifetimes should therefore be accompanied by held-out coverage, not merely by $R^{2}$ or MAE on a training set [25].

### 4.5. Small Data, Neural Networks, and Where the Effort Should Go

It is tempting to read the failure of our neural-network attempt (Section 3.12) as an argument that machine learning is inappropriate for coating degradation. That is not our conclusion. The literature contains genuine successes for polymer property prediction, but essentially all of them either operate on datasets larger by orders of magnitude or embed physics explicitly [9,10,11]. With 28 points and four composition levels, a network has neither the data to constrain its parameters nor an honest validation protocol available: with four compositions, LOCO leaves three training levels, and any random point-level split leaks the curve. The productive question is not “network or kinetic model” but “how much structure must be imposed for the available data to determine anything at all”. The present study answers that concretely for this system: a seven-parameter effective Weibull law identifies a composition trend; an eight-parameter two-process law does not identify its own mechanism; and an unconstrained network identifies nothing. Effort is better spent on measurements that break the identifiability ridge—early-time blend sampling, replicate gravimetry, and independent observation of PEG release (for example by quantifying PEG in the eluate)—than on model capacity.

### 4.6. Status of the Electrochemical Correlation

The monotone associations between the fitted degradation time scale and $i_{corr}$, $R_{p}$, $P_{e}$, and porosity are physically sensible and agree with the barrier-property arguments used for polymer-coated metals generally [29,30]. They are nonetheless exploratory for the three reasons set out in Section 3.11, of which the most important is confounding by composition: with a monotone $\tau_{eff}(c)$ and four compositions, correlation with $\tau_{eff}$ and correlation with c are indistinguishable. Establishing electrochemical measurement as a surrogate for gravimetric degradation—which would be genuinely valuable, since polarisation measurements take minutes rather than months—requires time-resolved polarisation data at matched immersion times, so that the electrochemical response can be regressed on accumulated mass loss within composition. We flag this as the single most cost-effective addition to a follow-up experimental campaign.

### 4.7. Limitations

Six limitations constrain every number in this paper. (i) The degradation data are axis-calibrated digitisations of a published raster figure, not raw measurements; the underlying replicate masses and standard deviations were not recoverable, so digitisation error and experimental noise are absorbed into a single scale parameter and cannot be separated. (ii) The design contains only four nominal compositions and a single laboratory, medium, and temperature; nothing here transfers automatically to other molecular weights, coating thicknesses, or flow regimes. (iii) The source publication contains internal numerical conflicts (Section 2.2) which we resolved by a documented rule; a different admissible rule would shift individual values by a few percentage points, though not the qualitative conclusions. (iv) The reported c=0.5 interpolation is a model output, not a measurement, and is unvalidated. (v) Extrapolation beyond 16 weeks, or outside 0≤c≤1, is entirely model-dependent and is not attempted. (vi) Because no new measurements were performed, several physicochemical descriptors that are known to modulate PCL and PCL/PEG hydrolysis are not available at the resolution of the individual mass-loss points reanalysed here. These include the local pH inside the coating (acid autocatalysis of ester hydrolysis by carboxylic-acid chain ends [5,28,29]), the initial crystallinity of the PCL phase (amorphous regions hydrolyse markedly faster than crystalline lamellae, so a shift in the amorphous/crystalline ratio changes the effective α and, more subtly, the shape parameter β [29,30]), the coating’s specific surface area and morphology (SEM evidence in the source paper [8] documents PEG-driven pore formation, but a quantitative surface-area value per composition is not reported), and the residual catalyst or moisture content of the commercial PCL/PEG starting materials, which can vary from batch to batch even for the same nominal supplier grade. All of these effects are ultimately absorbed into the calibrated Weibull parameters and the noise scale of the present model; they are compatible with, but not resolvable from, the data reanalysed here. A dedicated experimental follow-up combining pH tracking of the immersion medium, XRD-based crystallinity measurement, image-based surface-area characterisation, and end-group titration of the polymers would be needed to disentangle them, and is proposed in Section 4.8.

### 4.8. Physicochemical Mechanisms Not Resolved by the Reanalysed Dataset

PCL and PCL-blend-PEG degradation in aqueous media is governed by several coupled physicochemical processes. We summarise them here, together with the experimental characterisation already available in the source publication [8], so that the scope of the present statistical model is not misread. It should be emphasised at the outset that morphological, structural, chemical, and surface characterisations of the coatings analysed here are not missing from the record: SEM, GIXRD, FTIR and static-contact-angle wettability measurements were performed on all four compositions in the source study and are reported in Figures 3–5, 7 and 8 and Table 1 of Ref. [8]. The present manuscript is a Bayesian reanalysis of the mass-loss data of Figure 6 of [8], not a re-characterisation of the coatings, and it does not attempt to introduce these physicochemical parameters as explicit inputs to the kinetic model. Nevertheless, the mechanisms known to operate must be discussed, and this section does so, drawing on both the polymer-degradation literature and the experimental evidence provided in [8]. First, regarding acid autocatalysis and pH, PCL hydrolysis produces carboxylic-acid chain ends that can lower the local pH inside the coating and, in bulk devices, produce the classical “hollow-object” degradation pattern of aliphatic polyesters [5,28,29]. In the dip-coated films used in [8], the coating thickness measured by step profilometry ranges from 0.6 to 2 μm, and the immersion configuration is dynamic-flow SBF at a per-channel flow rate of 1.31 ± 0.04 mL min^−1^ [8]. The diffusion length for hydrolysis products in a film this thin is small compared with the length that would trap acid inside a bulk implant, and the flowing SBF continuously flushes the vicinity of the coating; the acid-autocatalytic hollow-object regime is therefore expected to be strongly attenuated in this system, even though it is not zero, and it contributes to the β > 1 signature reported for PCL in Table 4. Second, regarding morphology and the blend microstructure, SEM data in Figures 7 and 8 of [8] show that all pristine PCL and PCL-blend-PEG coatings exhibit an alveolar surface with characteristic cavity/hole populations, with the specific morphology varying strongly with composition. Pristine PCL and PCL-blend-PEG (3:1) present a PCL-continuous phase in which PEG is dispersed; after 8 weeks of dynamic immersion, holes of 140 nm to 3 μm appear in pristine PCL and holes up to 3.5 μm in PCL-blend-PEG (3:1), reaching 240 nm to 4.5 μm in PCL at 16 weeks and being accompanied by cracks and partial delamination in the (3:1) blend at the same time-point [8]. In contrast, PCL-blend-PEG (1:3) exhibits a much more homogeneous laminar morphology; after immersion, holes and pits are replaced by irregular polymeric agglomerates in the 260 nm to 3.5 μm range. This composition-dependent morphology is qualitatively compatible with the physical picture represented by our Weibull-mixture model and provides a plausible interpretation of the observed kinetic heterogeneity. In the PCL-rich 3:1 blend, PEG dissolution, water ingress, and PCL hydrolysis are likely to be strongly coupled, whereas the more laminar morphology of the 1:3 blend may facilitate more uniform water access. These SEM observations support a composition-dependent interpretation of the degradation kinetics but do not independently validate the two-process decomposition assumed by the model. The pore-network evolution is documented qualitatively in [8]; a quantitative time-resolved specific surface area (image-based) is not, and is therefore not available as a model input. Third, regarding crystallinity, qualitative structural evidence concerning crystallinity is available from the GIXRD patterns reported in Figure 4 of [8], although quantitative, per-composition, and time-resolved crystalline fractions were not reported. PEG contributes a sharp peak at 2θ = 19.3° and a broader peak at 23.2°, while PCL exhibits the characteristic semicrystalline orthorhombic (110), (111), and (200) reflections at 21.5°, 22.1°, and 23.8°. The blend patterns contain contributions from both polymers, providing qualitative evidence that crystalline domains of both constituents remain present after mixing. Because amorphous PCL is expected to hydrolyse faster than crystalline PCL lamellae [29,30], crystallinity may contribute to the observed degradation kinetics. However, the present model does not include crystallinity as an explicit parameter because a quantitative, per-composition, and time-resolved crystalline fraction is not available; consequently, a crystallinity-specific contribution cannot be isolated from the present mass-loss data. Fourth, regarding wettability and surface area, static water-contact-angle values reported in Table 1 of [8] evolve monotonically from bare titanium (117.1 ± 1.5°) to PCL (74.6 ± 4.3°), PCL-blend-PEG (3:1) (62.1 ± 1.6°), PCL-blend-PEG (1:3) (36.2 ± 0.9°), and pure PEG (28.3 ± 3.2°). The hydrophilic gradient produced by increasing PEG content directly controls the rate of initial water uptake and therefore the effective time constant of blend PEG dissolution; the surface-area-to-volume ratio of a 0.6–2 μm film is intrinsically large, and image-based surface roughness or porosity per composition and time-point—which would refine this picture—was not tabulated in [8]. Fifth, regarding the source of PCL and PEG and residual catalytic species, the source publication specifies that PCL (M_n_ = 14,000 Da) and PEG (M_n_ = 6000 Da) were purchased from Sigma-Aldrich (Darmstadt, Germany) and used as received [8]. Commercial PCL is typically obtained by ring-opening polymerisation, which can leave trace catalytic residues (e.g., tin(II) 2-ethylhexanoate) in the ppm range; even sub-100 ppm residues can shift hydrolysis kinetics [30]. The residual catalyst content of the specific batches used in [8] is not disclosed there and is therefore not recoverable in the present reanalysis; its effect, together with any batch-specific initial acid-end concentration and initial water content of the polymer powders, is absorbed into the calibrated Weibull parameters and the noise scale of the present model. Sixth, on the appropriate scope of the model: the present manuscript does not claim to be a mechanistic degradation model; it presents and validates a Bayesian statistical framework for describing and forecasting the mass-loss curves of [8], with fully quantified uncertainty. Statistical predictive utility and mechanistic completeness are complementary rather than alternative goals; the honest conclusion is that the present model is empirical and its transferability outside the parameter space of [8] is unvalidated. A dedicated experimental follow-up, integrating longitudinal pH tracking of the immersion medium per composition, wide-angle XRD to quantify per-composition crystallinity before and after immersion, image-based quantification of the specific surface area over time, and end-group titration of the starting polymers, would be needed to convert the composition-dependent Weibull parameters reported here into fully mechanistically interpretable quantities; such a study is proposed here as the natural continuation of the modelling framework developed in the present paper.

## 5. Conclusions

We re-analysed the 16-week degradation of dip-coated PCL, PCL-blend-PEG, and PEG films on titanium with a fully prespecified Bayesian workflow, comparing a mechanistic two-process Weibull kinetic model against a parsimonious single effective-Weibull baseline under exact composition-held-out and future-time refitting.

1. Both models sampled cleanly (max $\hat{R}\leq 1.005$, zero divergences), yet the two-process model failed every predictive clause of the prespecified decision rule: pooled LOCO MAE/RMSE of 43.7/48.3 pp against 37.6/40.9 pp for the baseline, two of four fold wins instead of three, and pooled temporal errors worse by 12–19%. The additional mechanistic process does not earn its extra parameter on these data.
2. Mechanistic separation is not identifiable here: $\alpha_{PEG}$ and $\gamma_{PEG}$ correlate at r=0.956, and five of eight parameters trigger identifiability cautions. Clean convergence diagnostics did not detect this, which argues for routine parameter-level identifiability reporting.
3. Neither model is adequate for an unmeasured composition. Empirical coverage of nominal 90% held-out intervals was 21.4% (two-process) and 3.6% (baseline); the baseline is the less-poor comparator, not a validated kinetic law.
4. The failure is diagnostic rather than merely negative. Composition-wise residuals show that PEG release inside a blend is matrix-retarded rather than instantaneous, which falsifies the parallel-independent-process assumption and identifies the coupling that a future model must include.
5. Time extrapolation is tractable where composition extrapolation is not (pooled MAE 13.2 versus 43.7 pp), so the binding constraint is the number of composition levels—not the length of the immersion campaign.
6. Predictive performance should improve substantially with a purpose-designed dataset: five to seven composition levels, early-time (hours-to-days) sampling of the blends, replicate gravimetry with reported standard deviations, and independent quantification of PEG in the eluate. The exploratory correlation between the fitted degradation time scale and the electrochemical parameters ($\rho_{S}=-1.00$ for $i_{corr}$, +1.00 for protective efficiency, exact p=0.083, n=4) is promising enough to justify time-resolved polarisation measurements in that campaign.

We report this negative result in full, with its diagnostics, code, and data, because an unvalidated predictor of implant coating lifetime is worse than an explicit statement of what current data can and cannot support.

## Figures and Tables

**Table 1:** Prior specification for the two-process Weibull model. Time scales are in hours; sigmoid denotes the logistic function, so $\beta_{j}\in (0.25,4.0)$ by construction. Baseline priors are analogous to $\alpha_{eff}$ replacing $\alpha_{PCL}$. The values quoted in this table are the 90% prior credible intervals (5–95 percentiles) of each parameter’s marginal prior. Posterior uncertainty is reported using highest-density intervals (HDIs), with the interval level stated explicitly in the corresponding table and text. Predictive calibration is assessed using nominal 50% and 90% predictive intervals.

Parameter	Prior	Physical Meaning
$\alpha_{PEG}$	Normal(log 12, 2)	log PEG dissolution time scale at c=0.5
$\alpha_{PCL}$	Normal(log 3000, 2)	log PCL hydrolysis time scale at c=0.5
$\gamma_{PEG},\gamma_{PCL}$	Normal(0, 2)	composition sensitivity of each time scale
$\eta_{\beta ,j}$	Normal(0, 1.5)	latent shape, $\beta_{j}=0.25+3.75\;sigmoid(\eta_{\beta ,j})$
σ	HalfNormal(5)	observation scale (percentage points)
ν−2	Exponential(0.1)	Student-*t* tail heaviness, ν>2

**Table 2:** Audited degradation dataset (n=28). Time is reported in the original experimental unit and in hours as used by the models. c is the PCL mass fraction. All values are axis-calibrated marker-centre digitisations of Figure 6 of [8] with an estimated reading uncertainty of ±0.3 pp (±0.5 pp for overlapping blend markers).

Composition	c	Time	Unit	Time (h)	Mass Loss (%)
PCL	1.00	2	weeks	336	2.74
PCL	1.00	4	weeks	672	4.61
PCL	1.00	8	weeks	1344	7.68
PCL	1.00	10	weeks	1680	9.87
PCL	1.00	12	weeks	2016	13.82
PCL	1.00	14	weeks	2352	17.54
PCL	1.00	16	weeks	2688	20.94
PCL:PEG 3:1	0.75	2	weeks	336	17.00
PCL:PEG 3:1	0.75	4	weeks	672	21.05
PCL:PEG 3:1	0.75	8	weeks	1344	34.98
PCL:PEG 3:1	0.75	10	weeks	1680	47.04
PCL:PEG 3:1	0.75	12	weeks	2016	58.88
PCL:PEG 3:1	0.75	14	weeks	2352	70.94
PCL:PEG 3:1	0.75	16	weeks	2688	76.54
PCL:PEG 1:3	0.25	2	weeks	336	15.90
PCL:PEG 1:3	0.25	4	weeks	672	31.47
PCL:PEG 1:3	0.25	8	weeks	1344	51.97
PCL:PEG 1:3	0.25	10	weeks	1680	58.77
PCL:PEG 1:3	0.25	12	weeks	2016	66.67
PCL:PEG 1:3	0.25	14	weeks	2352	73.36
PCL:PEG 1:3	0.25	16	weeks	2688	77.08
PEG	0.00	1	min	0.0167	25.96
PEG	0.00	3	min	0.0500	34.74
PEG	0.00	9	min	0.1500	60.79
PEG	0.00	12	min	0.2000	72.98
PEG	0.00	48	min	0.8000	92.98
PEG	0.00	60	min	1.0000	94.04
PEG	0.00	120	min	2.0000	95.44

**Table 3:** MCMC diagnostics for the full-data fits. Acceptance thresholds: $\hat{R}<1.01$, bulk and tail ESS ≥400, zero divergences, no tree-depth saturation, BFMI ≥0.30.

Model	Chains	Draws/Chain	Max $\hat{R}$	Min Bulk ESS	Min Tail ESS	Divergences	Tree-Depth Saturation	Min BFMI	Pass
Two-process Weibull	4	2000	1.005	954	1719	0	0	0.681	yes
Effective-Weibull baseline	4	2000	1.002	1577	1869	0	0	0.788	yes

**Table 4:** Posterior summaries (8000 draws). HDI = highest-density interval. The 3% and 97% HDI bounds correspond to a 94% highest-density interval. Time scales are in hours. Derived quantities are reported on their natural scale.

Model	Parameter	Mean	SD	3% HDI	97% HDI	bulk ESS	$\hat{R}$
Two-process	$\alpha_{PEG}$	−1.006	0.963	−2.887	0.712	2505	1.002
Two-process	$\gamma_{PEG}$	1.777	1.870	−1.641	5.363	2460	1.002
Two-process	$\alpha_{PCL}$	8.500	1.029	6.948	10.456	954	1.005
Two-process	$\gamma_{PCL}$	0.538	1.322	−1.742	3.227	1434	1.002
Two-process	$\beta_{PEG}$	0.992	0.592	0.358	2.192	5290	1.000
Two-process	$\beta_{PCL}$	2.320	0.876	0.854	3.830	2876	1.002
Two-process	σ (pp)	11.713	2.869	6.553	17.173	3019	1.000
Two-process	ν	3.527	2.970	2.000	7.110	3999	1.000
Baseline	$\alpha_{pure\;PEG}$	−1.877	0.232	−2.325	−1.429	5259	1.000
Baseline	$\beta_{pure\;PEG}$	0.836	0.388	0.409	1.463	3988	1.001
Baseline	$\tau_{pure\;PEG}$ (h)	0.157	0.037	0.088	0.224	5259	1.000
Baseline	$\alpha_{eff}$	7.878	0.187	7.574	8.232	1577	1.002
Baseline	$\gamma_{eff}$	1.809	0.530	0.902	2.785	2146	1.002
Baseline	$\beta_{eff}$	1.203	0.381	0.634	1.856	1874	1.001
Baseline	σ (pp)	9.781	2.474	4.954	14.118	1861	1.002
Baseline	ν	8.825	8.911	2.000	25.264	2530	1.001

**Table 5:** In-sample posterior-predictive residual summary by composition (n=7 per cell, percentage points). A positive mean residual indicates under-prediction of mass loss.

**Model**	**Composition**	**Mean Residual**	**MAE**	**RMSE**	**Max abs.**
Two-process	PEG (c = 0)	+2.92	3.46	4.10	8.00
Two-process	PCL-blend-PEG 1:3 (c = 0.25)	−26.06	26.06	31.77	59.06
Two-process	PCL-blend-PEG 3:1 (c = 0.75)	+10.11	14.70	17.19	27.70
Two-process	PCL (c = 1)	−3.76	3.76	3.99	6.23
Baseline	PEG (c = 0)	+2.05	2.73	3.36	7.28
Baseline	PCL-blend-PEG 1:3 (c = 0.25)	−2.13	2.48	2.72	3.95
Baseline	PCL-blend-PEG 3:1 (c = 0.75)	+17.43	17.43	19.60	29.45
Baseline	PCL (c = 1)	−9.19	9.19	9.45	11.55

**Table 6:** Exact leave-one-composition-out results (n=7 observations per fold). Folds A and D are boundary extrapolations; B and C are composition interpolations. Errors in percentage points; Max = maximum absolute error; Endpt. = error at the 16-week endpoint; LPD = posterior-mean log predictive density; C50 and C90 = empirical coverage of the nominal 50% and 90% predictive intervals; W90 = mean width of the 90% interval (pp). The pooled LPD row shows the same value (−8.95) for both models: this is not a numerical error but a genuine tie to two decimal places. The pooled LPD is dominated by a handful of held-out observations across the LOCO folds at which the tail heaviness of both predictive distributions is comparable. The fold-specific LPD values are not tied and provide the more informative comparison; the pooled row is retained for completeness only.

Model	Fold	Held-Out c	Type	MAE	RMSE	Max	Endpt.	LPD	C50	C90	W90
Two-process	A	0	extrap.	65.59	70.83	92.89	92.89	−13.24	0.00	0.00	6.67
Baseline	A	0	extrap.	50.82	55.20	72.26	66.12	−5.93	0.00	0.14	57.12
Two-process	B	0.25	interp.	38.49	41.18	61.41	19.33	−10.91	0.00	0.00	11.98
Baseline	B	0.25	interp.	40.85	44.40	66.17	19.50	−9.90	0.00	0.00	13.68
Two-process	C	0.75	interp.	22.00	26.54	44.37	44.37	−5.27	0.14	0.57	38.53
Baseline	C	0.75	interp.	26.44	29.20	43.71	43.71	−10.54	0.00	0.00	9.26
Two-process	D	1	extrap.	48.89	54.84	69.78	68.11	−6.37	0.00	0.29	48.40
Baseline	D	1	extrap.	32.22	34.87	45.50	45.50	−9.44	0.00	0.00	18.74
**Two-process**	**pooled**	—	—	**43.74**	**48.35**	—	—	−8.95	0.04	**0.214**	**26.39**
**Baseline**	**pooled**	—	—	**37.58**	**40.92**	—	—	−8.95	0.00	**0.036**	**24.70**

Bold indicates the pooled rows, which aggregate all 28 held-out observations across the four folds.

**Table 7:** Exact future-time forecasting results. PCL-containing coatings were fitted through 8 weeks (1344 h) and PEG through 60 min; the exact later observations were then predicted. Endpt. = error at the last observed time; LPD = posterior-mean log predictive density; C90 and W90 = empirical coverage and mean width of the nominal 90% predictive interval.

Model	Held-Out c	Cut-Off (h)	n	MAE	RMSE	Endpt.	LPD	C90	W90
Two-process	1	1344	4	2.03	2.38	3.35	−3.70	1.00	45.21
Two-process	0.75	1344	4	32.36	33.85	43.47	−5.80	0.25	48.76
Two-process	0.25	1344	4	7.20	9.41	0.74	−3.82	1.00	41.27
Two-process	0	1	1	5.74	5.74	5.74	−2.70	1.00	30.56
Baseline	1	1344	4	11.21	11.22	10.76	−3.81	1.00	36.02
Baseline	0.75	1344	4	25.47	26.26	32.03	−5.76	0.25	38.27
Baseline	0.25	1344	4	1.35	1.51	1.80	−3.34	1.00	40.45
Baseline	0	1	1	1.67	1.67	1.67	−2.42	1.00	16.20
**Two-process**	pooled	—	13	**13.24**	**14.48**	—	−4.31	0.77	43.96
**Baseline**	pooled	—	13	**11.83**	**12.13**	—	−4.16	0.77	36.55

Bold indicates the pooled rows, which aggregate all 13 held-out future-time observations.

**Table 8:** Evaluation of the prespecified model-selection rule. All clauses must pass for the two-process model to be preferred.

Clause	Requirement	Observed	Pass
Convergence	clean diagnostics for both models	max $\hat{R}$ 1.005/1.002, 0 divergences	yes
LOCO fold wins	≥3 of 4 on MAE	2 of 4	no
LOCO MAE improvement	≥+5%	−16.4%	no
LOCO RMSE improvement	≥+5%	−18.2%	no
Temporal error	must not worsen	MAE −11.9%, RMSE −19.5%	no
Identifiability	no required |r|>0.90	1 of 18 relationships at r=0.956	no
**Decision**	—	—	**not supported**

Bold indicates the overall decision row.

**Table 9:** Identifiability screening. Correlations are computed over 8000 posterior draws; the flag threshold is |r|>0.90.

Model	Relationships Screened	Breaching |r|>0.90	Largest |r|	Parameters with ≥1 Caution
Two-process Weibull	18	1 ($\alpha_{PEG}$ vs. $\gamma_{PEG}$)	0.956	5 of 8
Effective-Weibull baseline	11	0	0.673	2 of 7

**Table 10:** Prior and likelihood sensitivity analyses. MAE is in-sample and in percentage points. Variants failing diagnostics contribute no robustness evidence.

Variant	Likelihood	MAE	RMSE	Max $\hat{R}$	Min Bulk ESS	Diagnostics	Status
Default (primary)	truncated Student-*t*	12.32	17.71	1.005	954	pass	reported
Narrow α (SD 1)	truncated Student-*t*	12.54	17.64	1.006	612	pass	reported
Wide α (SD 3)	truncated Student-*t*	14.03	19.41	1.737	6	**fail**	excluded
Positive half-normal slopes	truncated Student-*t*	12.16	17.64	1.008	463	pass	reported
Non-truncated likelihood	Student-*t*	11.63	18.67	1.004	1195	pass	reported
Narrow β prior (SD 1)	truncated Student-*t*	12.45	17.76	1.006	466	pass	reported
Exclude provisional data	truncated Student-*t*	—	—	—	—	—	not estimable

Bold indicates the variant that failed the convergence diagnostics and was excluded from all robustness claims.

**Table 11:** Electrochemical parameters of the four coatings (mean ± SD, from Table 2 of [8]) alongside the model-derived degradation descriptors. Exploratory Spearman rank correlations against ${log}_{10}\tau_{eff}$, with exact permutation *p*-values over all 24 rank assignments (n=4; minimum attainable p=0.083), are $\rho_{S}=+0.80$ (p=0.333) for $E_{corr}$, −1.00 (p=0.083) for $i_{corr}$, +0.80 (p=0.333) for $R_{p}$, +1.00 (p=0.083) for $P_{e}$, and −0.80 (p=0.333) for P.

Coating	$E_{corr}$ (mV)	$i_{corr}$ (nA cm^−2^)	$R_{p}$ (Ω cm^2^)	$P_{e}$ (%)	P (%)	$\tau_{eff}$ (h)	$\beta_{eff}$
PEG	−317 ± 9.42	83.18 ± 1.81	552.7 ± 14.7	7.10 ± 1.05	72.95 ± 1.98	0.16	0.84
PCL:PEG 1:3	−356 ± 2.27	72.26 ± 1.05	486.0 ± 16.4	18.69 ± 0.94	82.35 ± 2.14	1678	1.20
PCL:PEG 3:1	−307 ± 3.56	60.32 ± 1.27	620.5 ± 18.3	32.31 ± 1.45	60.85 ± 1.47	4146	1.20
PCL	−292 ± 4.92	41.06 ± 1.38	1169.9 ± 66.1	54.28 ± 1.75	30.33 ± 1.27	6516	1.20

## Data Availability

The original contributions presented in this study are included in the article and Supplementary Materials. The audited dataset, complete modelling and validation code, posterior summaries, diagnostic tables, and all scripts required to regenerate every figure and table are openly available at https://github.com/sneakyevil96/prediction-model (accessed on 10 August 2026). The underlying immersion, gravimetric, and electrochemical measurements were reported previously [8].

## Supplementary Materials

The following supporting information can be downloaded at https://www.mdpi.com/article/10.3390/polym18182237/s1: Figure S1: Digitisation audit overlay of the selected marker centres on the source raster figure; Figure S2: Prior predictive simulations on linear and logarithmic time axes; Figure S3: Trace, rank and pair plots for both models; Table S1: Complete candidate-by-candidate data audit including all rejected notebook literals and prose values; Table S2: Convergence diagnostics for all exact LOCO, temporal and sensitivity refits.

## Abbreviations

The following abbreviations are used in this manuscript:

**Abbreviation**	**Meaning**
BFMI	Bayesian fraction of missing information
ESS	Effective sample size
HDI	Highest-density interval
LOCO	Leave-one-composition-out
LPD	Log predictive density
MAE	Mean absolute error
MCMC	Markov chain Monte Carlo
NUTS	No-U-turn sampler
PCL	Poly(epsilon-caprolactone)
PEG	Poly(ethylene glycol)
RMSE	Root-mean-square error
SBF	Simulated body fluid
pp	Percentage points
