# Supplementary material for: Bayesian Two-Process Weibull Modelling of PCL-Blend-PEG Coating Degradation in Simulated Body Fluid: Composition-Held-Out Validation, Identifiability Limits and Exploratory Electrochemical Correlation
Source: Polymers (Basel). 2026 Sep 14;18(18):2237. doi: 10.3390/polym18182237 (PMC13611490; doi:10.3390/polym18182237)
Supplement: Supplementary file 1 [file polymers-18-02237-s001.zip › polymers-4517234-supplementary.pdf]

Supplementary Materials

Bayesian two-process Weibull modelling of PCL-blend-PEG coating degradation in simulated body fluid

Figure S1 Digitisation audit

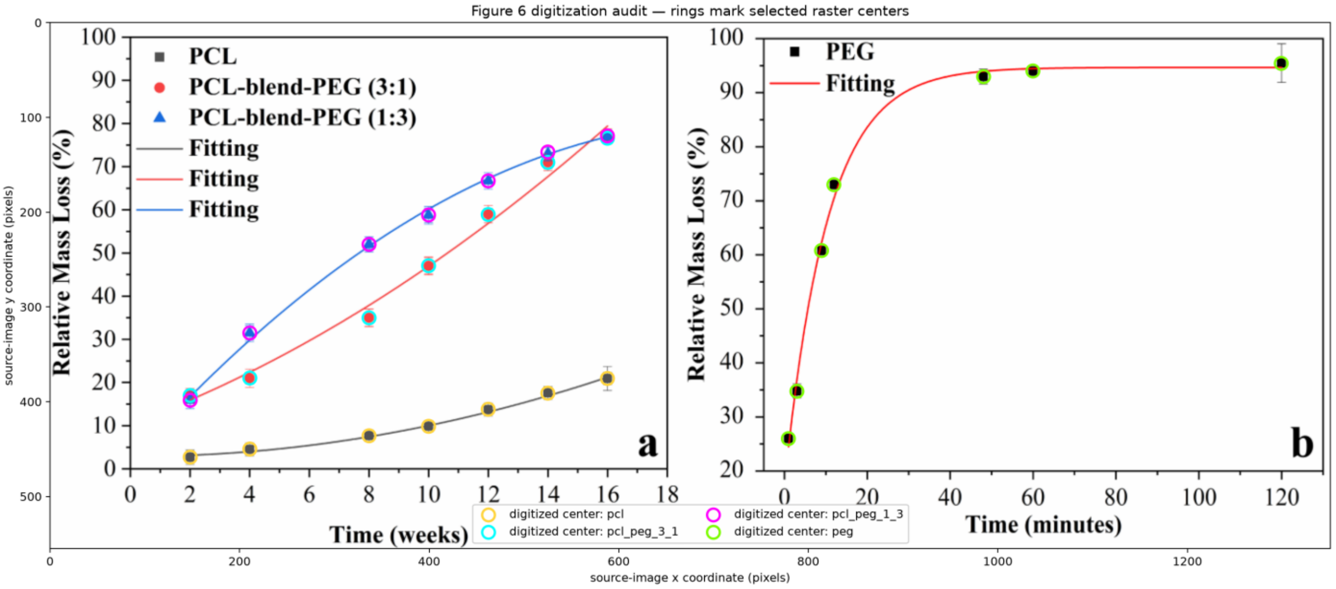

Figure S1. Selected marker centres overlaid on the original Figure 6 raster: (a) pure PCL and PCL:PEG blends over 16 weeks; (b) pure PEG over 120 min. Coloured rings identify all 28 centres used for inference. The outer axes show zero-based pixel coordinates measured from the top-left corner of the source raster. Source curves and error bars are reproduced as printed; they are not new model fits.

Calibration and uncertainty

For panel (a), weeks = 18(x - 85)/566 and mass loss (%) = 100(471 - y)/456. For panel (b), minutes = -177.3513 + 0.2288511x and mass loss (%) = 20 + 80(473 - y)/456. The panel (b) time calibration has a tick-fit RMS error of 0.06 min. Nominal marker times are retained in Table S1; time in hours equals weeks × 168 or minutes / 60.

All selected observations are provisional digitised values. Practical centre-reading uncertainty is approximately ±0.3 percentage points, increasing to ±0.5 for overlapping blend markers. These estimates describe graph reading, not experimental standard deviations; observation-level standard deviations were not supplied to the likelihood.

Source: Figure 6, publication p. 9, polymers-12-00717-v2.pdf (reference [8] in the main manuscript). The archived raster is 1351 × 555 pixels. Coordinates and calibration are also provided in Figure_S1_digitization_metadata.json.

Figure S2 Prior predictive simulations

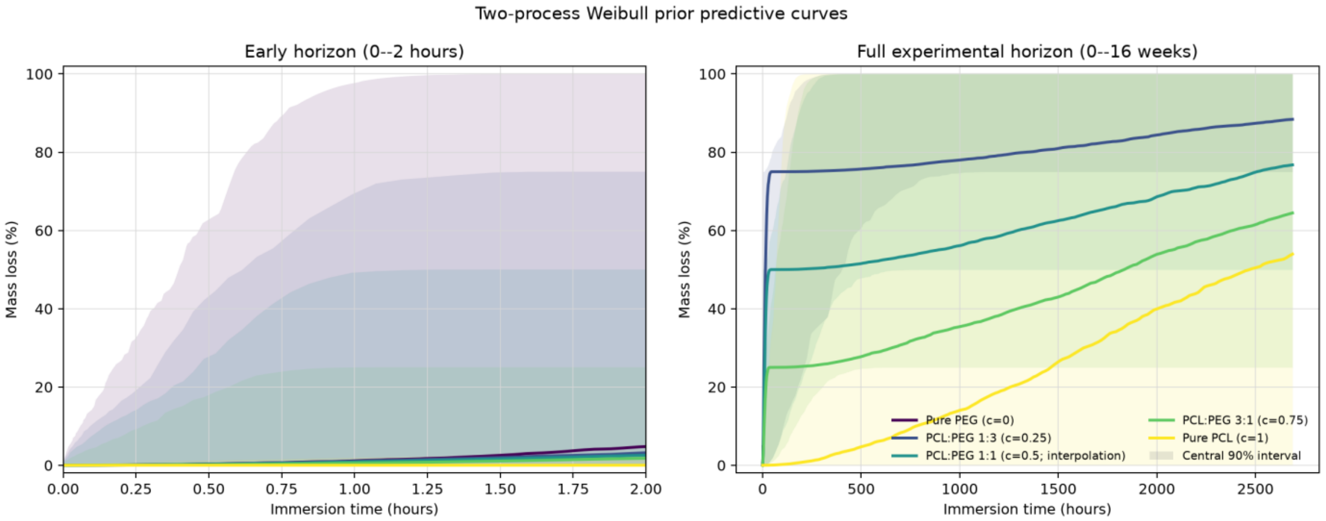

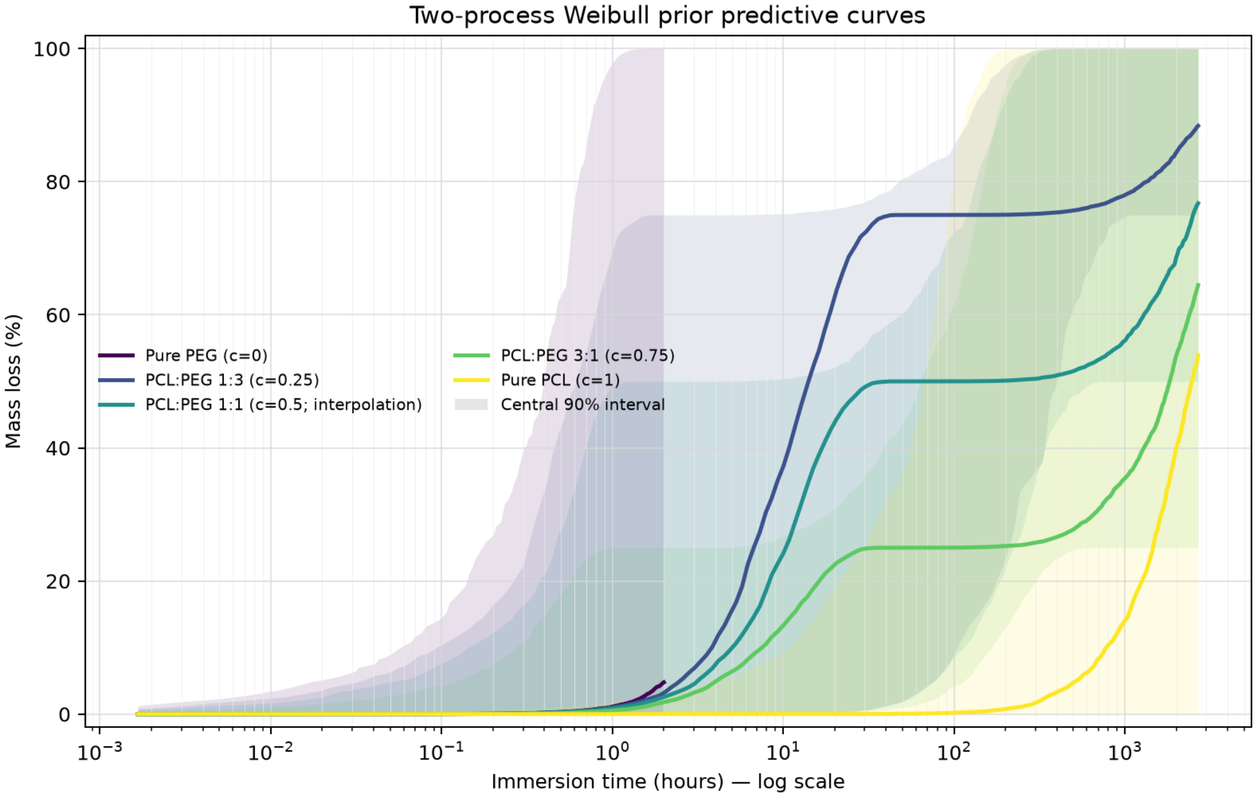

Figure S2. Prior simulation of latent mass-loss curves for the two-process Weibull model. (a) Linear time axes show the early 0-2 h interval and the full 0-16 week interval. (b) Logarithmic time axes show composition-specific curves. Lines are pointwise medians and shaded regions are central 90% intervals from 1,000 prior draws. c denotes PCL mass fraction; c = 0.50 is an unmeasured intermediate composition. Zero time is omitted on logarithmic axes.

The archived simulation evaluates the latent mean mass-loss function under prior parameter draws; it does not add Student-t observation noise. Thus, these bands describe prior uncertainty in the underlying curves rather than the full prior predictive distribution of measured mass loss. Only the two-process model was included in this archived prior check.

Figure S3a Two-process Weibull trace plots

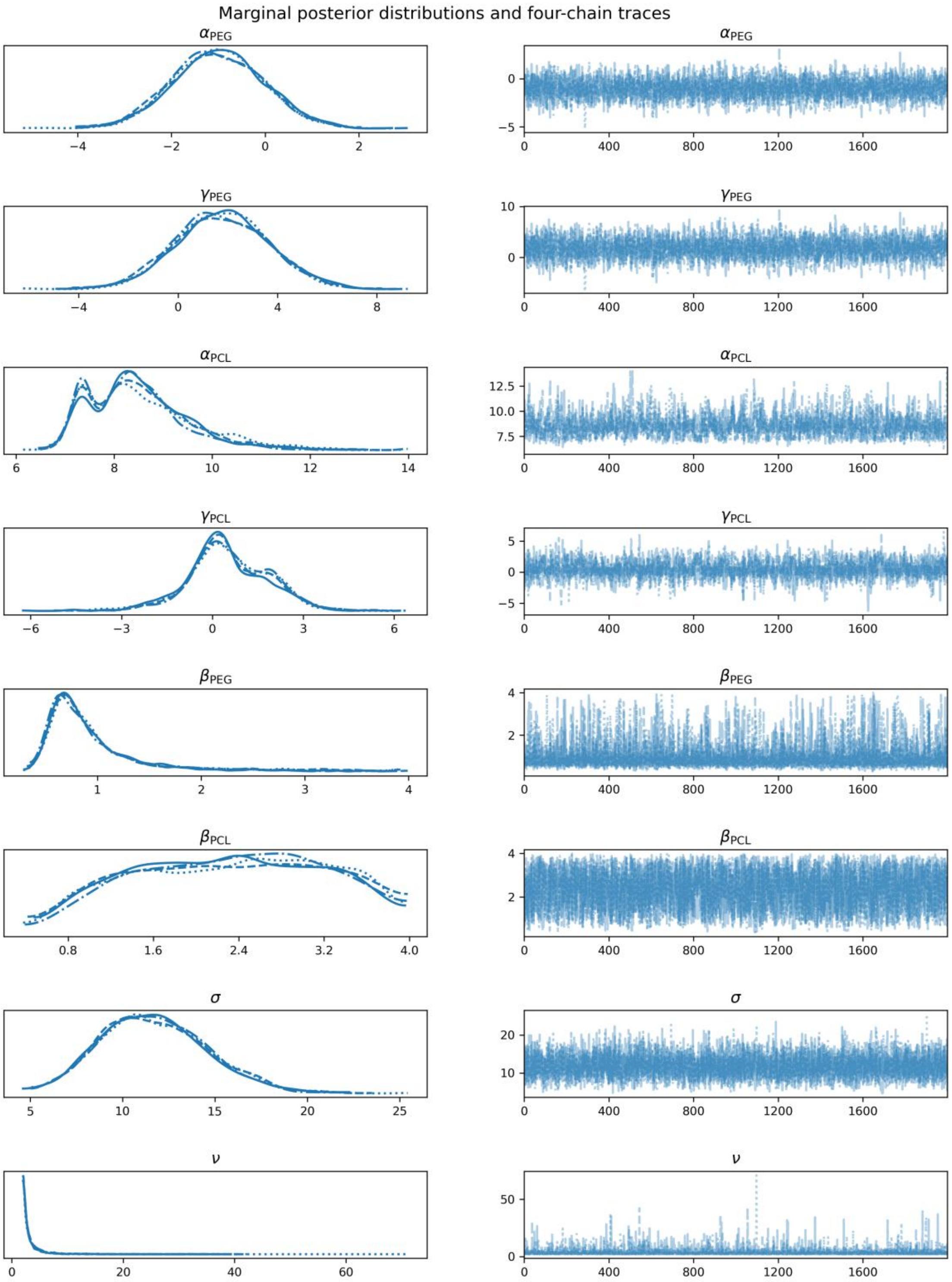

Figure S3a. Two-process Weibull, full-data fit (28 observations). Left columns show marginal posterior distributions; right columns show retained draws by chain. Each fit contains four chains with 2,000 retained draws per chain after 2,000 tuning iterations. Tuning draws are excluded.

Figure S3b Two-process Weibull rank plots

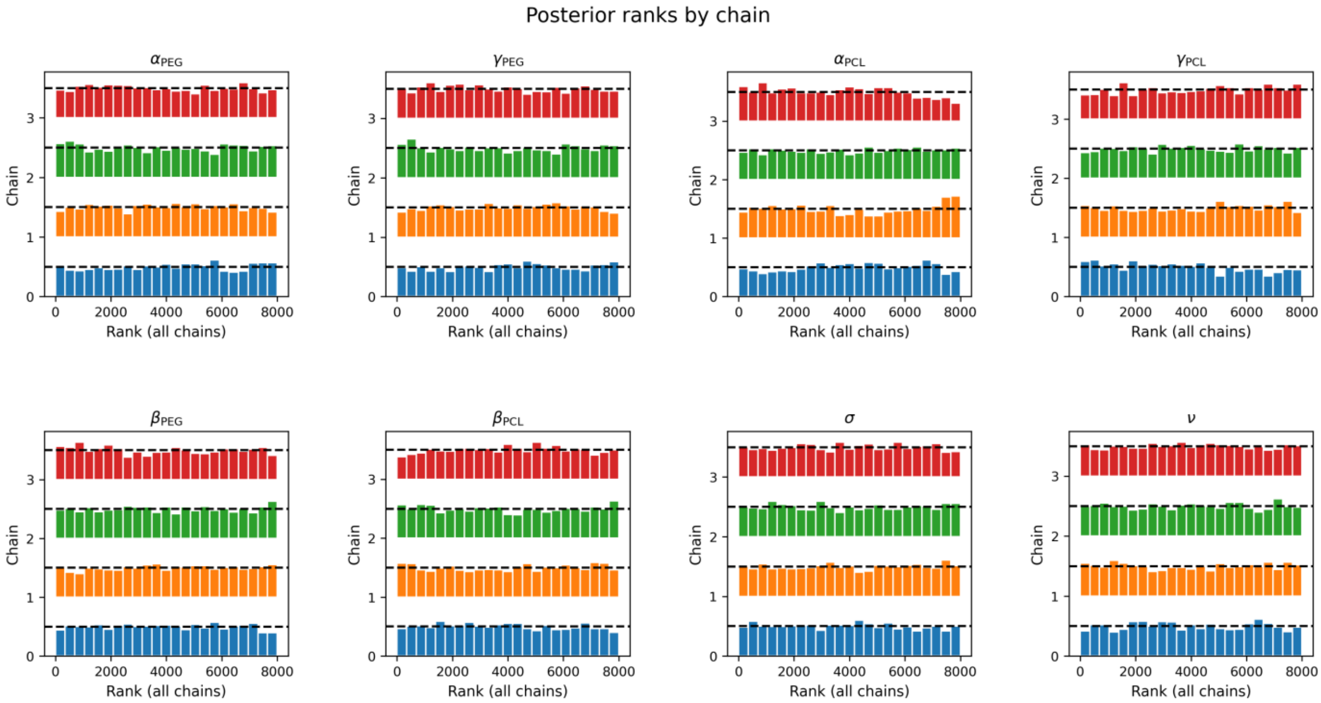

Figure S3b. Two-process Weibull, full-data fit (28 observations). Rank histograms compare the four chains for each displayed parameter. Chains with similar rank distributions support satisfactory mixing; numerical convergence criteria are reported in Table S2.

Figure S3c Two-process Weibull pair plots

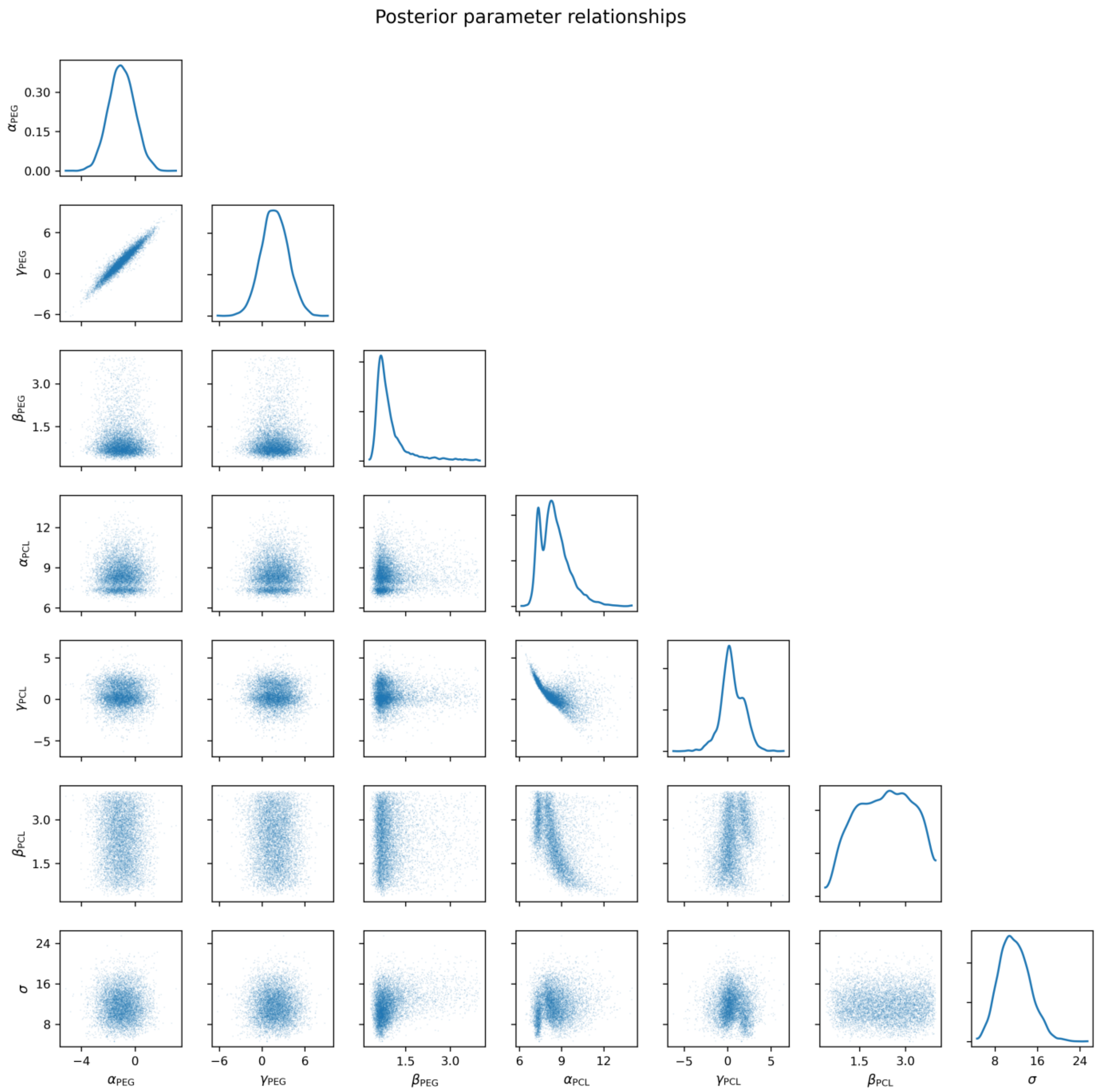

Figure S3c. Two-process Weibull, full-data fit (28 observations). Scatter panels show pairwise posterior relationships; diagonal panels show marginal distributions. Pair plots display the kinetic parameters and observation scale selected by the analysis script. The Student-t degrees of freedom are included in the trace and rank diagnostics but are not included in this selected pair matrix.

Strong dependence between the PEG intercept and composition slope remains visible despite satisfactory sampling diagnostics. Convergence does not establish separate identification of the two kinetic processes.

Figure S3d Effective-Weibull baseline trace plots

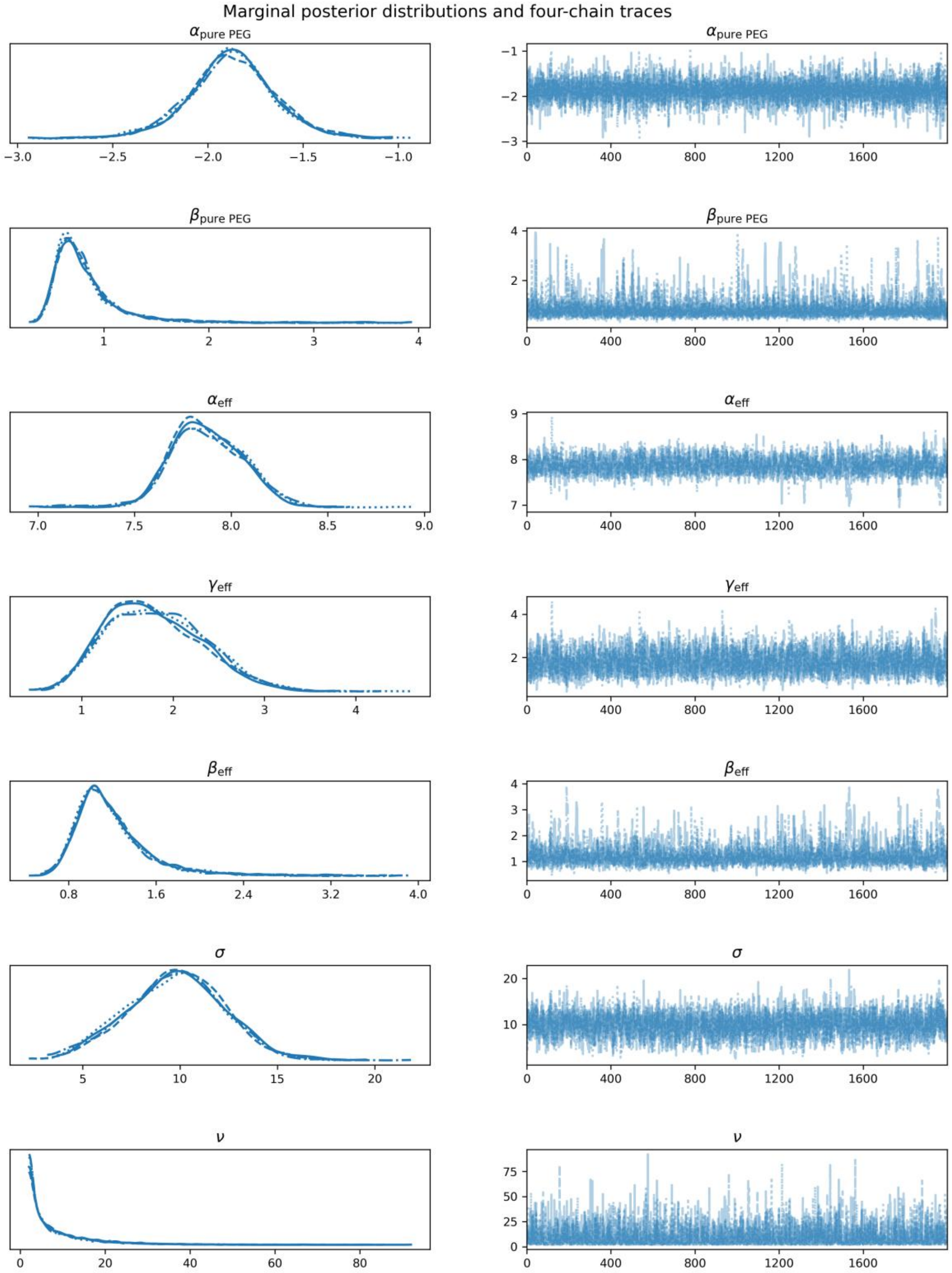

Figure S3d. Effective-Weibull baseline, full-data fit (28 observations). Left columns show marginal posterior distributions; right columns show retained draws by chain. Each fit contains four chains with 2,000 retained draws per chain after 2,000 tuning iterations. Tuning draws are excluded.

Figure S3e Effective-Weibull baseline rank plots

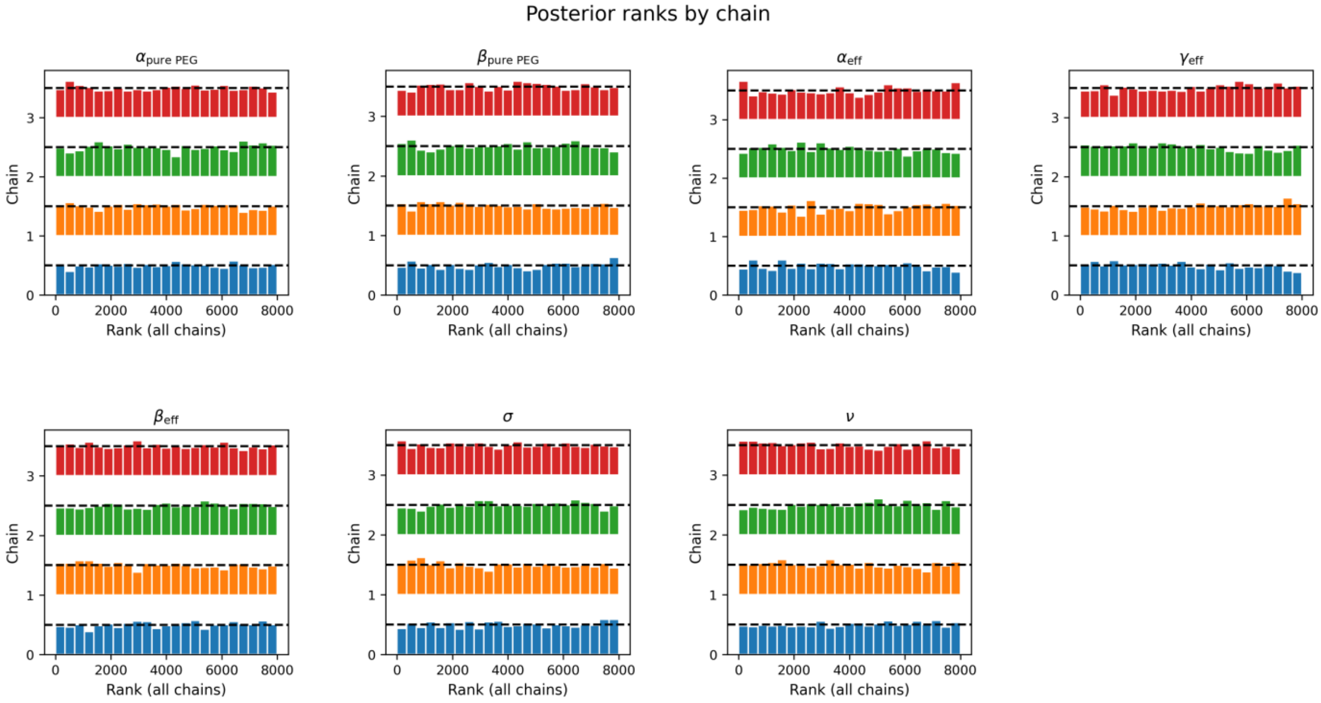

Figure S3e. Effective-Weibull baseline, full-data fit (28 observations). Rank histograms compare the four chains for each displayed parameter. Chains with similar rank distributions support satisfactory mixing; numerical convergence criteria are reported in Table S2.

Figure S3f Effective-Weibull baseline pair plots

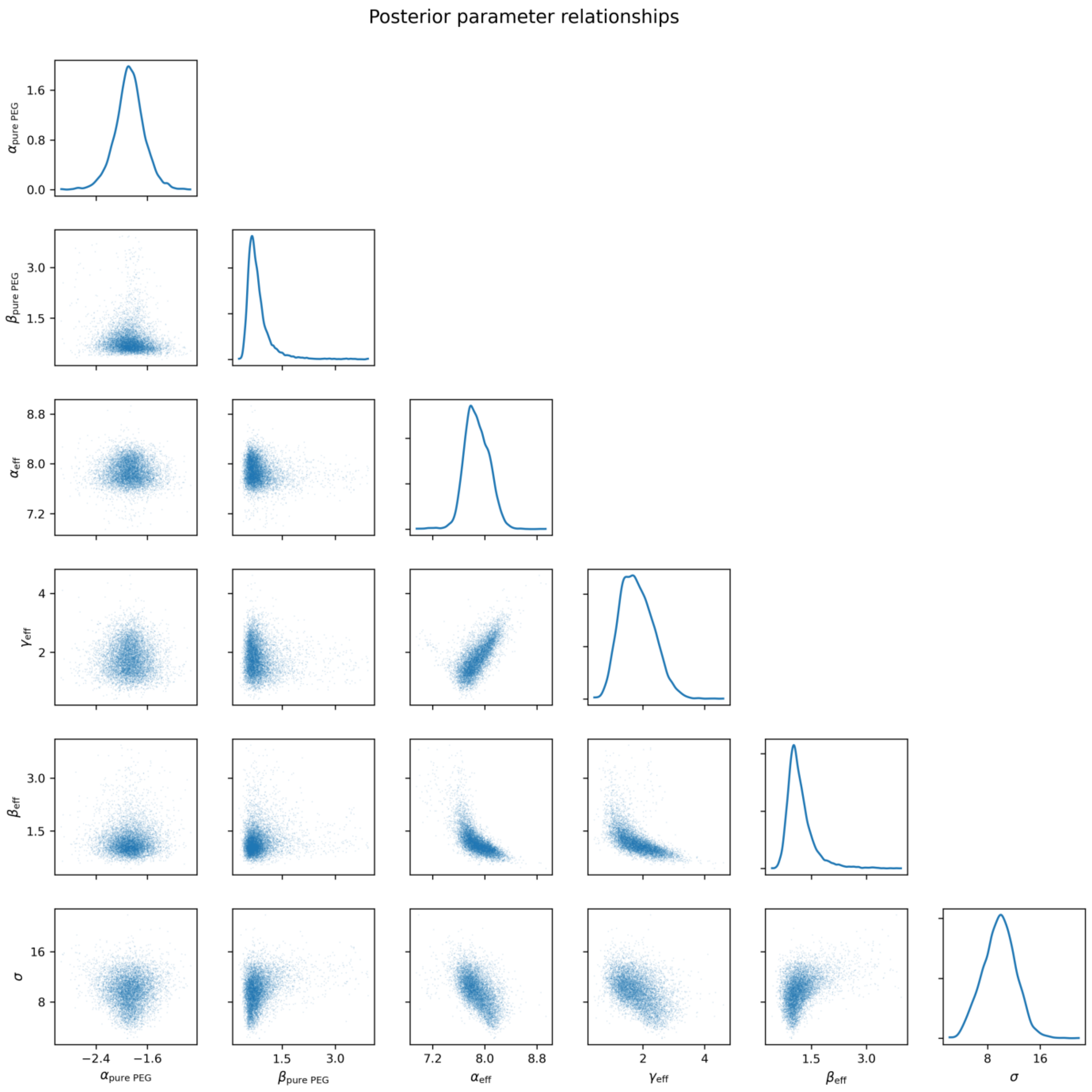

Figure S3f. Effective-Weibull baseline, full-data fit (28 observations). Scatter panels show pairwise posterior relationships; diagonal panels show marginal distributions. Pair plots display the kinetic parameters and observation scale selected by the analysis script. The Student-t degrees of freedom are included in the trace and rank diagnostics but are not included in this selected pair matrix.

Table S1 Complete candidate data audit

Table S1a. All 28 selected Figure 6 marker centres. Each row is provisional. Loss and centre-reading uncertainty are in percentage points; coordinates are source-image pixels. Blend labels give PCL:PEG mass ratios. Display rounding does not alter the machine-readable values.

ID	Material	Time	Hours	Loss (%)	x (px)	y (px)	Reading
F01	PCL	2 wk	336	2.741	147.9	458.5	±0.3
F02	PCL	4 wk	672	4.605	210.8	450.0	±0.3
F03	PCL	8 wk	1344	7.675	336.6	436.0	±0.3
F04	PCL	10 wk	1680	9.868	399.4	426.0	±0.3
F05	PCL	12 wk	2016	13.816	462.3	408.0	±0.3
F06	PCL	14 wk	2352	17.544	525.2	391.0	±0.3
F07	PCL	16 wk	2688	20.943	588.1	375.5	±0.3
F08	3:1	2 wk	336	16.996	147.9	393.5	±0.5
F09	3:1	4 wk	672	21.053	210.8	375.0	±0.3
F10	3:1	8 wk	1344	34.978	336.6	311.5	±0.3
F11	3:1	10 wk	1680	47.039	399.4	256.5	±0.3
F12	3:1	12 wk	2016	58.882	462.3	202.5	±0.3
F13	3:1	14 wk	2352	70.943	525.2	147.5	±0.3
F14	3:1	16 wk	2688	76.535	588.1	122.0	±0.5
F15	1:3	2 wk	336	15.899	147.9	398.5	±0.5
F16	1:3	4 wk	672	31.469	210.8	327.5	±0.3
F17	1:3	8 wk	1344	51.974	336.6	234.0	±0.3
F18	1:3	10 wk	1680	58.772	399.4	203.0	±0.3
F19	1:3	12 wk	2016	66.667	462.3	167.0	±0.3
F20	1:3	14 wk	2352	73.355	525.2	136.5	±0.3
F21	1:3	16 wk	2688	77.083	588.1	119.5	±0.5
F22	PEG	1 min	0.0166667	25.965	779.0	439.0	±0.3
F23	PEG	3 min	0.05	34.737	788.0	389.0	±0.3
F24	PEG	9 min	0.15	60.789	814.0	240.5	±0.3
F25	PEG	12 min	0.2	72.982	827.0	171.0	±0.3
F26	PEG	48 min	0.8	92.982	985.0	57.0	±0.3
F27	PEG	60 min	1	94.035	1037.0	51.0	±0.3
F28	PEG	120 min	2	95.439	1299.0	43.0	±0.3

Decision S: selected as the axis-calibrated plotted marker centre. All figure candidates originate from publication p. 9, Figure 6a (PCL and blends) or Figure 6b (PEG). Source-file and coordinate metadata are retained in the accompanying CSV and JSON files.

The printed source mass-change expression $[(m_f - m_i)/m_i] \times 100\%$ has the opposite sign to the positive plotted loss curves. The analysis interprets loss as $[(m_i - m_f)/m_i] \times 100\%$ and records the discrepancy.

Table S1 Rejected and nonselected candidates

Table S1b. Six prose candidates and the first 15 notebook literals. Difference = candidate minus selected marker-centre value at the same composition and time. “Almost 82” remains an approximate prose value; its difference uses 82 as a reference.

ID	Material	Stated time	Candidate loss (%)	Selected loss (%)	Difference (pp)	Code
P01	3:1	2 wk	17	16.996	+0.004	P
P02	3:1	16 wk	almost 82	76.535	+5.465	P
P03	PCL	4 wk	4.5	4.605	-0.105	P
P04	PCL	12 wk	13.7	13.816	-0.116	P
P05	PEG	48 min	93	92.982	+0.018	P
P06	PEG	120 min	98	95.439	+2.561	P
N01	PCL	336 h	4.5	2.741	+1.759	N
N02	PCL	672 h	8.2	4.605	+3.595	N
N03	PCL	1344 h	13.7	7.675	+6.025	N
N04	PCL	2016 h	22.5	13.816	+8.684	N
N05	PCL	2688 h	82.0	20.943	+61.057	N
N06	3:1	336 h	17.0	16.996	+0.004	N
N07	3:1	672 h	30.0	21.053	+8.947	N
N08	3:1	1344 h	45.0	34.978	+10.022	N
N09	3:1	2016 h	65.0	58.882	+6.118	N
N10	3:1	2688 h	82.0	76.535	+5.465	N
N11	1:3	336 h	32.0	15.899	+16.101	N
N12	1:3	672 h	52.0	31.469	+20.531	N
N13	1:3	1344 h	75.0	51.974	+23.026	N
N14	1:3	2016 h	90.0	66.667	+23.333	N
N15	1:3	2688 h	98.0	77.083	+20.917	N

Decision P: retained as a prose cross-check; no substitution into the uniformly digitised dataset. Decision N: rejected notebook literal lacking traceable raw-data provenance. Some literals are close to a selected marker by rounding; rejection does not imply that every literal differs materially from its corresponding marker.

Prose locations: P01-P04, publication p. 9, paragraph below Figure 6; P05-P06, p. 10, first paragraph. Notebook input cell 1: N01-N05, lines 67-75 (pclTimp, pclPierdere); N06-N10, lines 85-93 (bl31Timp, bl31Pierdere); N11-N15, lines 103-111 (bl13Timp, bl13Pierdere).

Table S1 Additional notebook candidates

Table S1c. Remaining seven notebook literals and four additional endpoint candidates. A dash denotes no selected observation at the same stated composition and time; no interpolation was used to create a comparison value.

ID	Material	Stated time	Candidate loss (%)	Selected loss (%)	Difference (pp)	Code
N16	PEG	10 min	40.0	—	—	N
N17	PEG	20 min	60.0	—	—	N
N18	PEG	30 min	75.0	—	—	N
N19	PEG	48 min	85.0	92.982	-7.982	N
N20	PEG	60 min	93.0	94.035	-1.035	N
N21	PEG	90 min	96.0	—	—	N
N22	PEG	120 min	98.0	95.439	+2.561	N
E01	PCL	16 wk	82	20.943	+61.057	E
E02	3:1	16 wk	82	76.535	+5.465	E
E03	1:3	16 wk	98	77.083	+20.917	E
E04	PEG	16 wk	100	—	—	T

N16-N22: notebook input cell 1, lines 123-138 (pegTimp, pegPierdere). E01-E04: input cell 2, lines 3804 onward, dateElectro field Degr_16sapt. All notebook locations refer to ANN degradare PEG_PCL art Polymers 2020.nb.

Decision N: notebook literal rejected for lack of traceable raw-data provenance. Decision E: additional notebook endpoint rejected; the PCL and blend entries repeat suspect endpoint assignments. Decision T: rejected endpoint whose 16-week label is incompatible with the PEG experiment ending at 120 min.

The four endpoint candidates are listed separately even when they repeat an earlier numerical literal, because they occur in a different notebook input cell. The empty notebook degradare PEG_PCL art Polymers 2020.nb contributes no candidates.

Audit provenance

The 60 entries comprise 28 selected raster values, six nonselected prose values, 22 rejected first-cell notebook literals, and four rejected second-cell endpoints. Prose and notebook candidates and their original locations are transcribed from the archived data audit and extraction code. The original PDF and Mathematica notebooks are not included in the supplied analysis folder; these candidate lists could therefore not be independently re-extracted for this supplement.

Table S2 Exact validation refit diagnostics

Table S2a. Convergence summaries for all eight exact leave-one-composition-out (LOCO) and two exact temporal refits. Full-data fits are included as references. Reported extrema cover the scalar parameters and scalar transformations in the archived diagnostic tables.

Fit	Model	Max $\hat{R}$	Min bulk ESS	Min tail ESS	Div.	Depth obs./limit	At limit	Min BFMI	Result
Full	TP	1.0049	953.6	1719.1	0	7/15	0	0.681	PASS
Full	EW	1.0025	1576.6	1869.0	0	5/15	0	0.788	PASS
LOCO A	TP	1.0022	1195.7	1330.0	0	7/15	0	0.732	PASS
LOCO A	EW	1.0011	1577.9	1237.4	0	5/15	0	0.892	PASS
LOCO B	TP	1.0024	2032.4	2460.9	0	7/15	0	0.799	PASS
LOCO B	EW	1.0027	1282.4	1719.3	0	6/15	0	0.796	PASS
LOCO C	TP	1.0038	1782.9	1567.4	0	7/15	0	0.617	PASS
LOCO C	EW	1.0033	2742.4	3141.0	0	5/15	0	0.837	PASS
LOCO D	TP	1.0025	2177.2	2211.1	0	6/15	0	0.777	PASS
LOCO D	EW	1.0015	4630.2	3183.1	0	4/15	0	0.873	PASS
TEMPORAL T	TP	1.0010	1750.3	2029.3	0	6/15	0	0.838	PASS
TEMPORAL T	EW	1.0016	1801.2	1303.4	0	6/15	0	0.656	PASS

TP = two-process Weibull; EW = effective-Weibull baseline. LOCO A, B, C and D hold out $c = 0, 0.25, 0.75$ and 1, respectively (21 training and seven held-out observations per fold). Temporal T trains on PCL-containing compositions through 8 weeks and PEG through 60 min (15 training and 13 held-out observations).

All rows above use four chains, 2,000 tuning iterations and 2,000 retained draws per chain, target acceptance 0.95 and maximum tree depth 15. BFMI is the minimum across chains. “At limit” counts transitions reaching the configured maximum depth.

Acceptance criteria: rank-normalised split $\hat{R} < 1.01$; bulk ESS ≥ 400 and tail ESS ≥ 400; zero divergences; zero maximum-tree-depth saturation; and BFMI ≥ 0.30 in every chain. Missing diagnostics are not classified as a pass.

Table S2 Sensitivity refit diagnostics

Table S2b. Final saved fits for all five fitted sensitivity variants of the two-process model. The wide-intercept-prior variant failed convergence and contributes no robustness evidence.

Fit	Model	Max $\hat{R}$	Min bulk ESS	Min tail ESS	Div.	Depth obs./limit	At limit	Min BFMI	Result
Narrow α	TP	1.0062	612.0	1332.3	0	6/15	0	0.682	PASS
Wide α	TP	1.7367	6.2	149.9	0	8/15	0	0.605	FAIL
Positive slopes	TP	1.0080	463.4	684.5	0	8/15	0	0.618	PASS
Untruncated t	TP	1.0040	1194.8	1091.6	0	6/15	0	0.706	PASS
Narrow ηβ	TP	1.0059	466.1	782.3	0	7/15	0	0.753	PASS

Variant	Prior or likelihood change	Tuning / retained per chain	Attempts
Narrow α	Both log-timescale intercept prior SDs: 2 → 1	1,000 / 1,000	1
Wide α	Both log-timescale intercept prior SDs: 2 → 3	3,000 / 1,000	2
Positive slopes	Normal slopes → positive half-normal slopes	3,000 / 1,000	2
Untruncated t	Remove Student-t likelihood truncation	1,000 / 1,000	1
Narrow ηβ	Both shape-transform prior SDs: 1.5 → 1	1,000 / 1,000	1

All sensitivity fits use four chains and maximum tree depth 15. Initial fits use target acceptance 0.95; retries use 0.99. Each final sensitivity fit contains 4,000 retained draws. “Narrow ηβ” changes the prior width on the unconstrained shape variable before its transformation to the Weibull shape parameter.

Initial attempts and non-estimable analysis

Analysis	Archived status
Wide α, initial attempt	A failed diagnostic check triggered a retry. The initial posterior and numerical diagnostic values were not retained.
Positive slopes, initial attempt	A failed diagnostic check triggered a retry. The initial posterior and numerical diagnostic values were not retained.
Exclude provisional observations	Not estimable: all 28 observations are provisional, leaving zero observations. No fit was performed.

The retry counts are recorded in prior_sensitivity.csv; the validation script replaces the initial in-memory fit and saves only the final attempt. No numerical values are imputed for the missing initial diagnostics. The accompanying Table_S2_parameter_diagnostics.csv supplies per-parameter $\hat{R}$, ESS and Monte Carlo standard errors for every saved validation and sensitivity refit, together with sampler diagnostics.
